# TREM2 is a receptor for non-glycosylated mycolic acids of mycobacteria that limits anti-mycobacterial macrophage activation

**DOI:** 10.1038/s41467-021-22620-3

**Published:** 2021-04-16

**Authors:** Ei’ichi Iizasa, Yasushi Chuma, Takayuki Uematsu, Mio Kubota, Hiroaki Kawaguchi, Masayuki Umemura, Kenji Toyonaga, Hideyasu Kiyohara, Ikuya Yano, Marco Colonna, Masahiko Sugita, Goro Matsuzaki, Sho Yamasaki, Hiroki Yoshida, Hiromitsu Hara

**Affiliations:** 1 Department of Immunology, Graduate School of Medical and Dental Sciences, Kagoshima University, Kagoshima, Japan; 2 Research and Development Department, Japan BCG Laboratory, Tokyo, Japan; 3 Biomedical Laboratory, Division of Biomedical Research, Kitasato University Medical Center, Kitamoto, Saitama Japan; 4 Division of Molecular and Cellular Immunoscience, Department of Biomolecular Sciences, Faculty of Medicine, Saga University, Saga, Japan; 5 Department of Hygiene and Health Promotion Medicine, Graduate School of Medical and Dental Sciences, Kagoshima University, Kagoshima, Japan; 6 Tropical Biosphere Research Center, University of the Ryukyus, Nishihara, Okinawa Japan; 7 Department of Molecular Immunology, Division of Host Defense, Research Institute for Microbial Disease, Osaka University, Osaka, Japan; 8 Faculty of Medicine, Osaka City University Graduate School of Medicine, Osaka, Japan; 9 Department of Pathology and Immunology, BJC Institute of Health at Washington University, St. Louis, MO USA; 10 Laboratory of Cell Regulation, Institute for Virus Research, Graduate School of Biostudies, Kyoto University, Kyoto, Japan

**Keywords:** Antimicrobial responses, Immune evasion, Monocytes and macrophages, Pattern recognition receptors

## Abstract

Mycobacterial cell-wall glycolipids elicit an anti-mycobacterial immune response via FcRγ-associated C-type lectin receptors, including Mincle, and caspase-recruitment domain family member 9 (CARD9). Additionally, mycobacteria harbor immuno-evasive cell-wall lipids associated with virulence and latency; however, a mechanism of action is unclear. Here, we show that the DAP12-associated triggering receptor expressed on myeloid cells 2 (TREM2) recognizes mycobacterial cell-wall mycolic acid (MA)-containing lipids and suggest a mechanism by which mycobacteria control host immunity via TREM2. Macrophages respond to glycosylated MA-containing lipids in a Mincle/FcRγ/CARD9-dependent manner to produce inflammatory cytokines and recruit inducible nitric oxide synthase (iNOS)-positive mycobactericidal macrophages. Conversely, macrophages respond to non-glycosylated MAs in a TREM2/DAP12-dependent but CARD9-independent manner to recruit iNOS-negative mycobacterium-permissive macrophages. Furthermore, TREM2 deletion enhances Mincle-induced macrophage activation in vitro and inflammation in vivo and accelerates the elimination of mycobacterial infection, suggesting that TREM2-DAP12 signaling counteracts Mincle-FcRγ-CARD9-mediated anti-mycobacterial immunity. Mycobacteria, therefore, harness TREM2 for immune evasion.

## Abstract (web summary)

Mycobacterial cell wall lipids can drive immunoevasion, but underlying mechanisms are incompletely understood. Here the authors show TREM2 is a pattern recognition receptor that binds non-glycosylated mycolic acid-containing lipids and inhibits Mincle-induced anti-mycobacterial macrophage responses.

## Introduction

Tuberculosis (TB) is a chronic infectious disease caused by *Mycobacterium tuberculosis* (Mtb), and remains a major cause of morbidity and mortality worldwide. Most infected individuals do not manifest clinical symptoms of TB (referred to as latent infection). In this phase, the bacteria are dormant with a nonreplicating phenotype and resistant to reactive intermediates and antibiotics, consistently evading host immune recognition^1^.

A distinctive trait of mycobacteria is their highly lipid-rich outer membrane, which is not only critical for their replication, acid-fast properties, and drug resistance, but also plays a key role in pathogenicity^2,3^ Some of these lipids trigger immunopathologic response, whereas others have immuno-evasive functions associated with virulence and latency^4^. Mycolic acids (MAs) are mycobacterium and related spp.-specific lipids with extremely long fatty acids (C_60_–C_90_) and represent predominant component in mycobacterial cell walls. MAs form the cell wall skeleton by covalently linking to the arabinogalactan (ABG) and peptidoglycan (PGN) basement layer, and also exist on the cell wall surface in either glycosylated or non-glycosylated forms^5^. Their composition is altered dynamically depending on mycobacterial life cycle stage and external environmental conditions^6,7^, and this affects bacterial virulence and latency by controlling host immunity. For example, glycosylated MA-containing lipids, such as trehalose dimycolate (TDM; also known as cord factor) and glucose monomycolate (GMM), possess potent pro-inflammatory and adjuvant activity and are capable of inducing lung granulomas when injected into animals^8,9^. These glycolipids are predominantly synthesized in actively replicating mycobacteria, while their levels are markedly diminished in dormant mycobacteria^6^. Non-glycosylated MA lipids, such as free MA (fMA) and glycerol monomycolate (GroMM), are associated with mycobacterial persistence, immune suppression, and biofilm formation. fMA is increased in cell walls in nonreplicating persistent mycobacteria^6^, forms biofilms^10,11^, and also inhibits cytokine production and phagosome–lysosome fusion in macrophages. A hypervirulent mutant Mtb strain (∆*mce1*) with a disruptied *mce1* operon, which results in excess accumulation of fMA in the cell wall^12,13^, induces a weak macrophage chemokine response^14^, fails to elicit a strong Th1 response, and causes poorly organized lung granulomas in mice^13^, relative to that of the wild-type (WT) strain. GroMM is associated with latent infection^15^, and induces eosinophilic inflammation and T helper (Th)2-type cytokine production^16^, which might counteract Th1-mediated antimycobacterial immunity. However, the molecular mechanisms by which these non-glycosylated MA lipids modulate host immunity remain unclear.

Although pathogen recognition by macrophages usually triggers the immune responses that eliminate the pathogens, macrophages are the primary target cells of Mtb and serve as an intracellular niche for Mtb propagation and latency^17^. Mtb has evolved remarkable immune evasion strategies that enable intracellular persistence in macrophages^18^. The cell wall lipids phthiocerol dimycocerosates (PDIMs) and the structurally related phenolic glycolipids (PGLs) represent virulence factors found only in a limited subset of pathogenic mycobacteria, such as hypervirulent W-Beijing strains of Mtb^19^. The surface expression of these lipids on mycobacteria masks ligands for toll-like receptors (TLRs) that are required for the induction of inducible nitric oxide synthase (iNOS)-positive M1-type microbicidal macrophages, and instead recruits iNOS-negative non-microbicidal macrophages (referred to as “permissive macrophages”) to the site of infection, thereby facilitating the intracellular Mtb survival and propagation^20^. The recruitment of permissive macrophages is dependent on CCR2 and the induction of its ligand monocyte chemoattractant protein-1 [MCP-1 (CCL2)] by PGL through activation of the STING cytosolic sensing pathway^20,21^.

Evidence for the importance of immunoreceptor tyrosine-based activation motif (ITAM)-coupled receptors, including C-type lectin receptors (CLRs) and immunoglobulin superfamily receptors, is accumulating with regard to innate immunity against a variety of pathogens^22–25^. These receptors associate with the ITAM-bearing signaling adaptors DAP12 or FcRγ, or harbor cytoplasmic ITAM-like motifs called hemITAM for signal transduction^26^. Ligand recognition by these receptors triggers tyrosine phosphorylation in ITAM, followed by activation of the tyrosine kinase Syk, leading to the downstream activation of mitogen-activated protein kinases and nuclear factor-kappa B through the signaling adaptor caspase recruitment domain family member 9 (CARD9). CARD9 acts in complex with B-cell lymphoma/leukemia-10 and mucosa-associated lymphoid tissue lymphoma translocation protein-1, and plays an essential role in ITAM-coupled receptor-induced myeloid cell activation^26,27^. Recent reports highlight the importance of CARD9 and the FcRγ-associated CLRs, which recognize mycobacterial cell wall glycolipids, in antimycobacterial innate immunity^28^. CARD9-deficient mice are highly susceptible to Mtb infection^29^. Mincle (Clec4e) recognizes TDM to elicit pulmonary inflammation and induces granuloma formation in an FcRγ-dependent manner^30^. MCL (Dectin-3/Clec4d) also recognizes TDM and form a dimer with Mincle to stabilize its surface expression^31,32^. Dectin-2 (Clecsf10) recognizes mannose-capped lipoarabinomannan (LAM), and plays a central role in production of IL-10 and IL-2 during mycobacterial infection^33^. DCAR (mouse Clec4b1) recognizes phosphatidylinositol mannosides, and promotes monocyte recruitments and the Th1 responses^34^. In contrast to CLR-associated FcRγ, the other ITAM-bearing signaling adaptor, DAP12, might negatively regulate the antimycobacterial immune response, because DAP12 deficiency accelerates the clearance of mycobacteria and the granuloma formation in lungs upon Mtb or *Mycobacterium bovis* Bacille de Calmette et Guerin (BCG) infection^35,36^. These observations implicate unknown DAP12-associated regulatory receptors that might possibly recognize immune-suppressive ligands in mycobacteria. However, these theoretical receptors, as well as the precise mechanisms of immune suppression via DAP12, have not been described.

In the present study, we present evidence suggesting that the DAP12-associated receptor triggering receptor expressed on macrophage 2 (TREM2) recognizes mycobacterial MA-containing lipids that are distinct from those recognized by Mincle and counteracts the Mincle–FcRγ–CARD9-mediated antimycobacterial immune response. We show that the glycosylated MA-containing lipids induce mycobactericidal macrophages in a Mincle/FcRγ/CARD9-dependent manner. Conversely, the non-glycosylated MA-containing lipids induce mycobacteria-permissive macrophages in a TREM2/DAP12-dependent but CARD9-independent manner. Moreover, loss of TREM2 markedly enhances Mincle-induced macrophage activation in vitro and inflammation in vivo, and accelerated the elimination of mycobacterial infection in mice. These results suggest that mycobacteria evade host immunity via TREM2 to avoid mycobactericidal macrophage activation through the Mincle–CARD9 pathway. This finding describes a mechanism by which mycobacteria controls the host immune response through a host regulatory innate immune receptor, which may have implications for treatment of mycobacteriosis, including TB.

## Results

### TREM2 recognizes mycobacteria

**Fig. 1:**
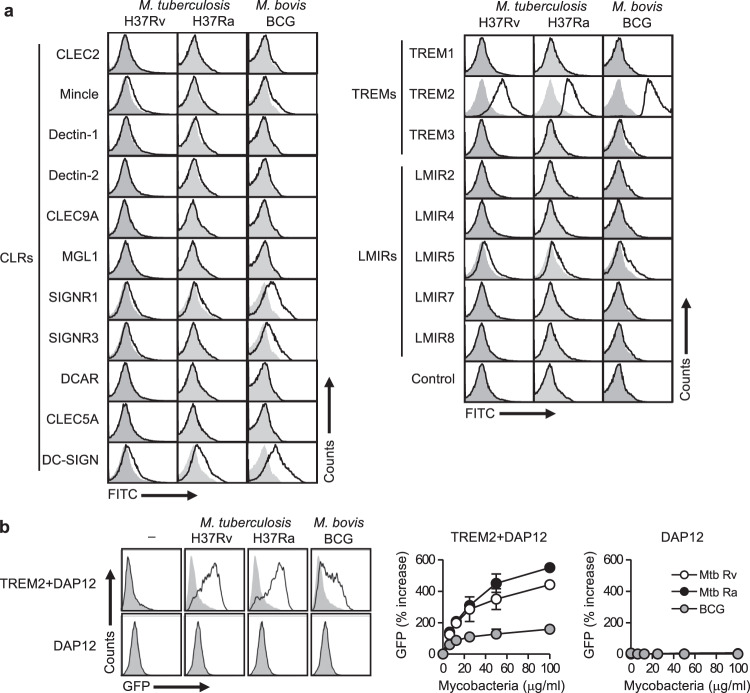
TREM2 recognizes mantihumanycobacteria. **a** Heat-killed Mtb H37Ra or H37Rv, or *M. bovis* BCG were incubated with the indicated CLR, TREM, and LMIR family receptors fused to the Fc-antibody fragment or with the control Fc fragment, followed by staining with antihuman IgG-FITC secondary antibody. The binding of each receptor-Fc protein was analyzed by flow cytometry. Open histograms show binding data for the indicated receptor-Fc proteins. The gray-filled histograms show background fluorescence of the control staining. **b** NFAT-GFP reporter cells expressing TREM2 + DAP12 or DAP12 alone were stimulated with the indicated amounts of heat-killed Mtb H37Ra or H37Rv, or *M. bovis* BCG for 24 h, followed by analysis of GFP fluorescence by flow cytometry. The histograms on the left indicate the data from stimulation with 100 μg/ml of the indicated mycobacteria. Data in the right panels are presented as percent increase over control values of unstimulated cells. Data are presented as the mean ± SEM of duplicate assays and representative of three independent experiments. Source data are provided as a Source data file.

To screen novel ITAM-coupled receptors capable of recognizing mycobacteria, we analyzed the binding of 20 known ITAM-coupled CLR, TREM, and leukocyte mono-Ig-like receptor (LMIR; CD300) family receptors^22,37–39^ fused to Fc-antibody fragments to heat-killed mycobacterial strains, including the virulent strain Mtb H37Rv, the attenuated strain Mtb H37Ra, and the vaccine strain *M. bovis* BCG, using flow cytometry. In addition to several CLRs previously reported to recognize mycobacteria, including Mincle, specific ICAM-3 grabbing nonintegrin-related (SIGNR)1, SINGNR3, and DC-SIGN^30,40^, this screening identified TREM2 and LMIR5 as novel receptors capable of binding to mycobacteria (Fig. 1a). Especially, TREM2 demonstrated a strong binding capacity to all tested mycobacterial strains, which prompted us to further characterize this interaction.

To investigate whether recognition of mycobacteria by TREM2 activates intracellular ITAM signaling, we used nuclear factor of activated T cells (NFAT)-driven green fluorescent protein (GFP) reporter cells (2B4)^30^ ectopically expressing TREM2 and its signaling subunit DAP12 (refs. ^41,42^). As reported previously^30^, Mtb H37Rv, Mtb H37Ra, and *M. bovis* BCG stimulated NFAT-GFP signaling in reporter cells expressing Mincle and FcRγ (Supplementary Fig. 1a). We found that all these mycobacterial strains clearly activated reporter cells expressing TREM2 and DAP12, but not in those expressing only DAP12, in a dose-dependent manner (Fig. 1b). Whereas, none of these strains activate reporter cells expressing TREM1 plus DAP12 (Supplementary Fig. 1b). Importantly, the level of stimulation by TREM2 and Mincle differed between the strains (Fig. 1b and Supplementary Fig. 1a), suggesting that TREM2 and Mincle recognize different ligands commonly expressed by these mycobacterial strains.

### TREM2 recognizes MAs

**Fig. 2:**
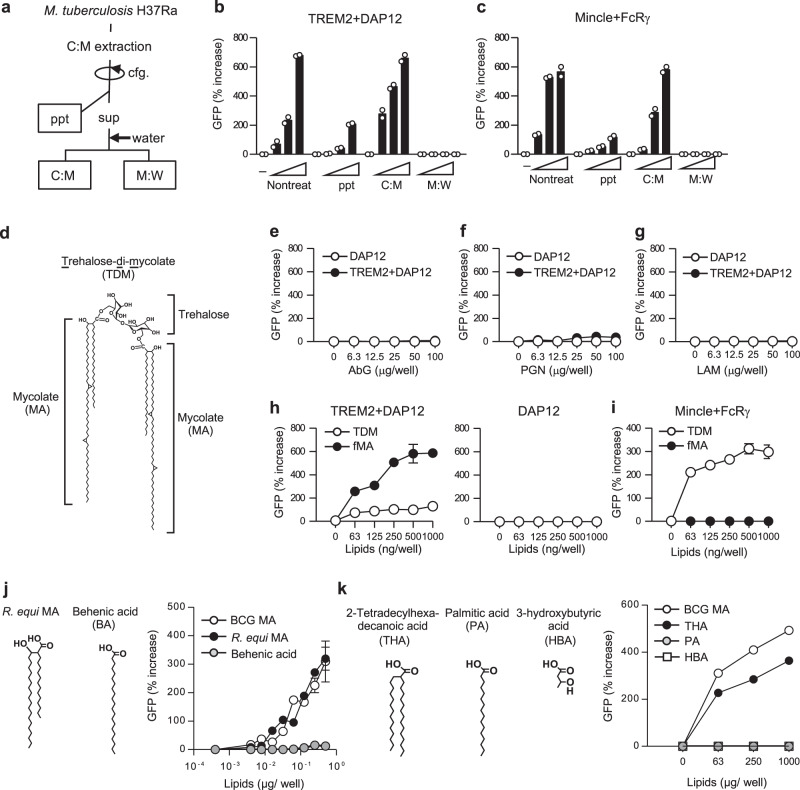
TREM2 recognizes MAs. **a** Schematic diagram of solvent-based de-lipidation and fractionation of Mtb H37Ra. Bacteria were de-lipidated by the treatment with chloroform/methanol. After centrifugation (cfg.), soluble extracts (sup) were mixed with water and separated into the lipid-soluble (C:M) and the water-soluble methanol:water (M:W) fractions. **b**, **c** NFAT-GFP reporter cells expressing TREM2 + DAP12 (**b**) or Mincle + FcRγ (**c**) were stimulated for 24 h with either untreated or de-lipidated bacteria (ppt) or plate-coated C:M or M:W fractions indicated in **a**, followed by analysis of GFP fluorescence by flow cytometry. **d** Chemical structure of TDM. The structure of α-mycolate-containing TDM is shown. **e**–**g** NFAT-GFP reporter cells expressing TREM2 + DAP12 or DAP12 alone were stimulated with the indicated amounts of ABG (**e**), PGN (**f**), and LAM (**g**) for 24 h, followed by analysis of GFP fluorescence by flow cytometry. Data are presented as percent increase over control values of unstimulated cells. **h**, **i** NFAT-GFP reporter cells expressing TREM2 + DAP12 or DAP12 only (**h**) or Mincle + FcRγ (**i**) were stimulated with the indicated amounts of TDM or fMA, and analyzed as described in **e**–**g**. **j**, **k** NFAT-GFP reporter cells expressing TREM2 + DAP12 were stimulated with the indicated amounts of BCG MA, *R. equi* MA, or BA (**j**) or THA, PA, or HBA (**k**) and analyzed as described in **e**–**g**. Data are presented as the mean ± SEM of duplicate (**b**, **c**, **e**–**i**, **j**) or triplicate (**k**) assays and representative of three independent experiments. Source data are provided as a Source data file.

TREM2 binding to mycobacteria implies that its ligand(s) exist on the cell wall surface, where mycobacteria express a wealth of unique lipids that influence the host immune responses^43^. Given that TREM2 binds to various endogenous mammalian lipids^44,45^, we first examined whether mycobacterial lipids contain TREM2 ligand(s). De-lipidation of Mtb H37Ra with chloroform and methanol (C:M; Fig. 2a) markedly diminished the level of stimulation in TREM2 reporter cells (Fig. 2b), as well as in Mincle reporter cells as reported^30^ (Fig. 2c). Accordingly, the lipid-containing C:M fraction (Fig. 2a), but not the hydrophilic M:W fraction showed strong stimulating activity in TREM2 reporter cells (Fig. 2b) as with Mincle reporter cells (Fig. 2c), implicating that TREM2 ligands were present in the cell wall lipid fraction as with so Mincle ligands^30^. We then next tested the TREM2-stimulating activity using known major constituents of mycobacterial cell wall, including the glycans PGN and ABG, the immunostimulatory glycolipids LAM and TDM (Fig. 2d), and fMA (Fig. 2d). We observed that ABG, PGN, and LAM did not activate the TREM2 reporter cells, even at higher concentrations (Fig. 2e–g), whereas we observed substantial ligand activity with TDM and fMA (Fig. 2h). Importantly, loss of trehalose moiety from TDM (i.e., fMA) markedly increased the TREM2-stimulating activity (Fig. 2h), while it completely abolished the Mincle-stimulating activity as reported (Fig. 2i)^30^. These results suggest that TREM2 recognizes the MA moiety of TDM, and that the sugar moiety interferes with this recognition.

We then investigated the structure of MA necessary for TREM2 recognition. Extremely long (C_60_–C_90_) and branched alkyl chains are hallmarks of mycobacterial MAs. To assess the importance of the length and branching, we compared the stimulatory activity of mycobacterial MA with that of *Rhodococcus equi* MA, which has shorter alkyl chains (C_35_), and of behenic acid (BA), which is a linear fatty acid with an alkyl chain (C_22_) of similar length with the mero-chain of *R. equi* MA (Fig. 2j). *R. equi* MA exhibited a similar level of stimulatory activity in TREM2 reporter cells as mycobacterial MA, whereas the level of stimulation with BA was very low, even at higher concentrations of BA (Fig. 2j), suggesting that the branched structure rather than the alkyl chain length was important for TREM2 recognition of MA. To further investigate this, we tested the ligand activity of palmitic acid (PA; C_16_), 2-tetradecylhexadecanoic acid (THA; C_14_/C_16_), which bears a PA-based synthetic fatty acid with similar branching structure as MA, but lacking its 3-hydroxyl residue, and 3-hydroxybutyric acid (HBA), which has only the carboxyl and hydroxyl residue at the branching moiety of MA (Fig. 2k). We detected TREM2 stimulation with THA, albeit at a lower level than MA, but could not detect stimulation with PA and HBA (Fig. 2k), suggesting that TREM2 recognition requires a branched fatty acid structure with an alkyl chain.

The R47H mutation in TREM2, which is associated with a higher risk of several neurodegenerative diseases^46–49^, impairs the recognition of brain lipids^45,50^. We found that the TREM2 R47H mutation also attenuated MA recognition (Supplementary Fig. 2), suggesting that this residue is important for MA recognition by TREM2.

### Contrasting recognition of MA-containing lipids by Mincle and TREM2

**Fig. 3:**
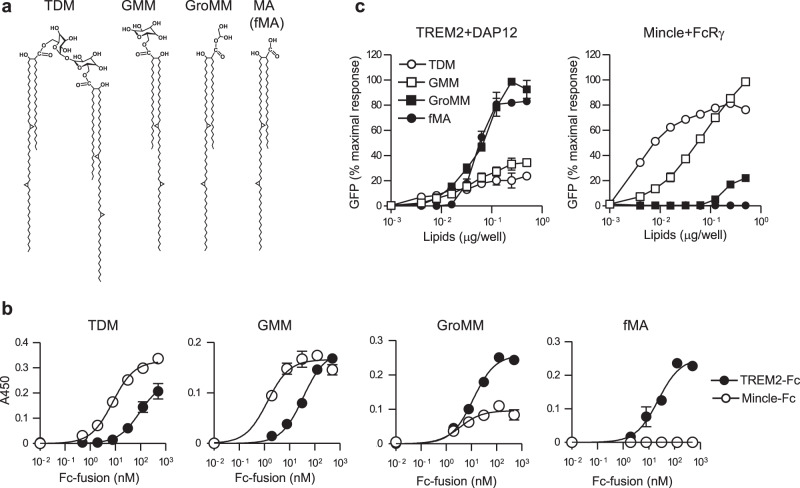
TREM2 and Mincle preferentially recognize distinct MA-containing lipids based on their glycosylation. **a** Chemical structure of glycosylated (TDM and GMM) and non-glycosylated (GroMM and fMA) MA-containing lipids found in Mtb. The structures of α-mycolate-containing lipids are included. **b** The indicated amounts of TREM2 or Mincle-Fc fusion proteins were added to wells coated with 0.5 μg/well TDM, GMM, GroMM, or fMA, followed by incubation with anti-human IgG-horseradish peroxidase and detection of bound proteins using an enzyme-linked immunosorbent assay (ELISA)-based colorimetric assay. **c** NFAT-GFP reporter cells expressing TREM2 + DAP12 or Mincle + FcRγ were stimulated with the indicated amounts of TDM, GMM, GroMM, or fMA coated on the plates for 24 h, followed by analysis of GFP fluorescence by flow cytometry. Values are plotted as percent maximal response of the largest value. Data are presented as mean ± SEM of duplicate assays and are representative of at least three independent experiments. Source data are provided as a Source data file.

Mycobacteria express a variety of MA-containing lipids on their cell wall^51^. Since both Mincle and TREM2 have the capacity to recognize MA-containing lipids, we next compared their binding activity to the glycosylated (TDM and GMM) or the non-glycosylated (GroMM and fMA) MA-containing lipids (Fig. 3a). using the receptor-Fc fusion proteins. We observed that Mincle-Fc not only showed strong binding to TDM as expected, but also binding to GMM, whereas it showed no detectable binding to fMA and weak binding to GroMM only at higher concentrations (Fig. 3b). By contrast, TREM2-Fc showed strong binding to GroMM and fMA, but showed relatively much weaker binding to TDM and GMM (Fig. 3b). We then examined whether the different binding of MA-containing lipids to TREM2 and Mincle reflect their receptor-stimulatory activities using NFAT-GFP reporter cells. Consistent with the binding data, TREM2 reporter cells strongly responded to MA and GroMM, but weakly to TDM and GMM, whereas Mincle reporter cells responded strongly to TDM and GMM, but weakly to GroMM only at higher concentrations and did not respond to fMA (Fig. 3c). Therefore, TREM2 and Mincle preferentially recognize non-glycosylated and glycosylated MA-containing lipids, respectively.

### Mincle and TREM2 induce distinct macrophage activation

**Fig. 4:**
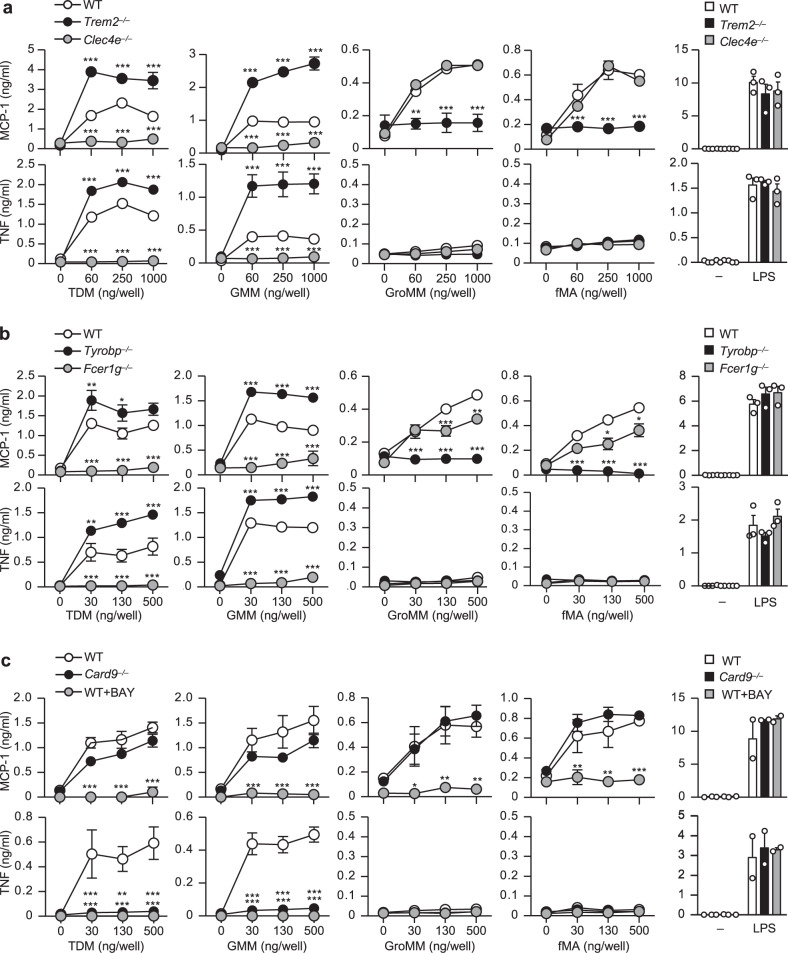
Distinct lipid recognition and macrophage activation through Mincle and TREM2. **a**–**c** Peritoneal macrophages from wild-type (WT), *Trem2*^*−/−*^, or *Clec4e*^*−/−*^ mice (**a**), WT, *Tyrobp*^*−/−*^, or *Fcer1g*^*−/−*^ mice (**b**), or WT or *Card9*^*−/−*^ mice (**c**) were stimulated with the indicated amounts of TDM, GMM, GroMM, or fMA coated on the plates or with LPS (100 ng/ml) for 24 h. WT cells were stimulated in the presence or absence of the Syk inhibitor BAY-613606 (BAY) (**c**). MCP-1 and TNF production in the culture supernatant was measured by ELISA. Data are presented as the mean ± SEM of triplicate assays and are representative of three independent experiments. The statistical significance was calculated by two-way ANOVA followed by Bonferroni’s test. *p < 0.05, **p < 0.01, ***p < 0.001. Source data are provided as a Source data file.

To investigate the relevance of TREM2 and Mincle recognition of MA-containing lipids in the activation of innate immune cells, we stimulated peritoneal macrophages from WT, TREM2-deficient (*Trem2*^*−/−*^), or Mincle-deficient (*Clec4e*^*−/−*^) mice with either glycosylated or non-glycosylated MA-containing lipids and examined their production of MCP-1, a pivotal monocyte chemoattractant implicated in TB pathology^52–54^, and TNF, an essential cytokine for granuloma formation and TB control^55,56^. Interestingly, we found that the activation of WT macrophages by the glycosylated MA-containing lipids TDM or GMM or by lipopolysaccharide (LPS) induced production of both MCP-1 and TNF, whereas activation of macrophages with the non-glycosylated MA-containing lipids GroMM or fMA induced production of MCP-1 but not TNF (Fig. 4a), as well as other pro-inflammatory cytokines, including IL-6 and IL-12p40 (Supplementary Fig. 3a). Loss of Mincle almost completely abolished TNF, as previously^30^, as well as MCP-1 in response to TDM as expectedly^30^. We found that Mincle deficiency also abolished these productions induced by GMM (Fig. 4a). However, Mincle deficiency did not affect MCP-1 production in response to GroMM or fMA. By contrast, loss of TREM2 almost completely abolished the MCP-1 production in response to GroMM or MA, but not to TDM or GMM (Fig. 4a). These results demonstrated that glycosylated and non-glycosylated MA-containing lipids elicited distinct macrophage activation, which was dependent on Mincle and TREM2, respectively.

Triggering of ITAM-coupled receptors on myeloid cells activates the ITAM–Syk–CARD9 signaling pathway to induce cytokine production^57^. To investigate whether MCP-1 and TNF production induced by MA-containing lipids depends on this pathway, we first confirmed the requirement for DAP12 and FcRγ, which are the ITAM-containing signaling subunits of TREM2 and Mincle, respectively. As expected, we observed that FcRγ-deficient (*Fcer1g*^*−/−*^) and DAP12-deficinet (*Tyrobp*^*−/−*^) macrophages almost phenocopied Mincle-deficient and TREM2-deficient macrophages, respectively, with similar patterns of defects in TNF and MCP-1 productions in response to glycosylated or non-glycosylated MA-containing lipids (Fig. 4b). However, FcRγ deficiency slightly dampened MCP-1 induction by fMA or GroMM at higher concentrations, implicating that FcRγ might partly contribute to TREM2 signaling. We then examined the requirement for Syk and CARD9 for each of these responses. Treatment of WT macrophages with a Syk inhibitor (BAY-613606) abrogated both MCP-1 and TNF production in response to all MA-containing lipids (Fig. 4c), indicating essential role for Syk in the response to these lipids. Intriguingly, although TNF production induced by glycosylated MAs through Mincle was abolished in CARD9-deficient (*Card9*^*−/−*^) macrophages, as expected^58^, CARD9-deficiency did not affect MCP-1 production induced by both glycosylated and non-glycosylated MA-containing lipids (Fig. 4c). Collectively, these results demonstrated that mycobacterial MA-containing lipids induced TNF production through Mincle via the canonical FcRγ–Syk–CARD9 pathway, while they induced MCP-1 production via the ITAM–Syk pathway, but independent of CARD9 signaling (Supplementary Fig. 3b).

### TREM2/DAP12 signal inhibits Mincle-induced macrophage activation

Interestingly, we observed that loss of TREM2 markedly enhanced MCP-1 and TNF production by peritoneal macrophages in response to TDM or GMM (Fig. 4a). This was also true in *Tyrobp*^*−/−*^ macrophages (Fig. 4b), suggesting an inhibitory role of TREM2/DAP12 signaling in Mincle/FcRγ-induced macrophage activation. We did not observe an increase in cytokine response when the same preparation of *Trem2*^*−/−*^ macrophages were stimulated with TLR ligands, such as Pam_3_CSK_4_ (for TLR2), LPS (for TLR4), Poly (I:C) (for TLR3), CpG-ODN (for TLR9), or with the Dectin-1 ligand zymozan (Fig. 4a and supplementary Fig. 4a, b). This selective enhancement of Mincle activation in the absence of TREM2 was more prominent in bone marrow-derived macrophages (BMDMs), where TDM-induced TNF was detected at very low levels in WT BMDMs. Nevertheless, *Trem2*^*−/−*^ or *Tyrobp*^*−/−*^ BMDMs showed substantial TNF production in response to TDM (Supplementary Fig. 4c), whereas this enhancement was not observed following Pam_3_CSK_4_ or LPS stimulation. In addition, we observed an increased cytokine response following stimulation of *Trem2*^*−/−*^ BMDMs with mycobacterial total lipids (C:M fraction in Fig. 2a) prepared from Mtb R37Ra or *M. bovis* BCG (Supplementary Fig. 4d). This response was largely dependent on Mincle, as *Clec4e*^*−/−*^ BMDMs produced markedly lower TNF in response to the total lipids compared to WT BMDMs (Supplementary Fig. 4d). These data clearly indicated that TREM2/DAP12 signaling selectively inhibited macrophage activation induced by Mincle–FcRγ signaling.

### Triggering of TREM2 induces permissive macrophages

**Fig. 5:**
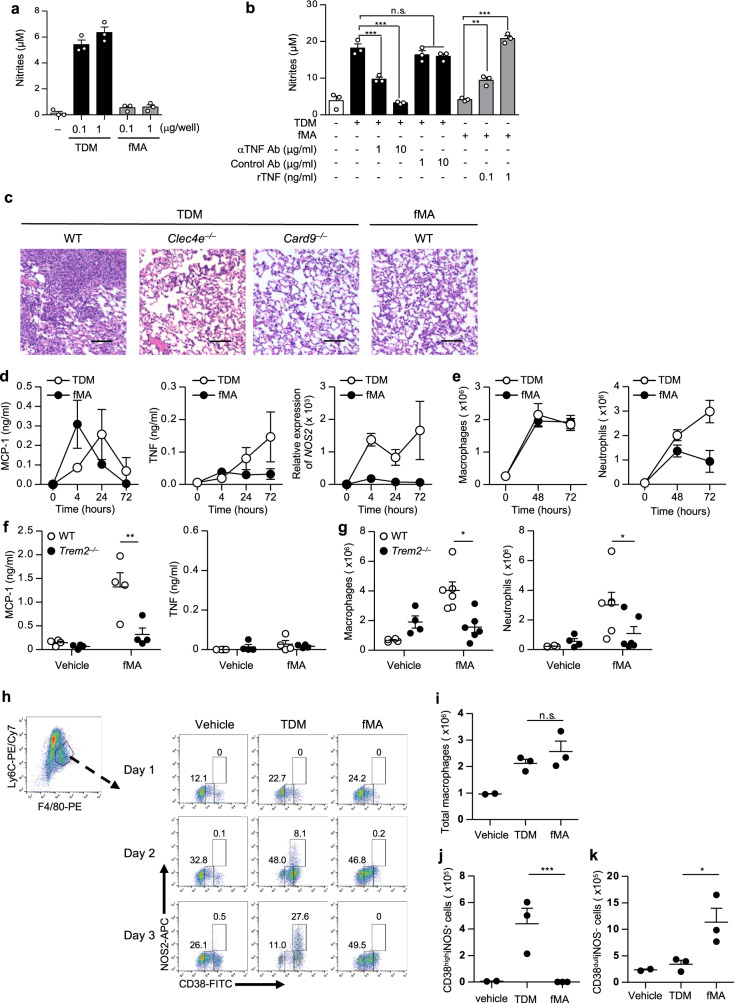
Triggering of TREM2 by MA induces the permissive macrophages. **a**, **b** BMDMs were stimulated for 24 h with the indicated amount of TDM or fMA in the presence of 10 ng/ml IFN-γ (**a**), 1 μg of TDM with or without the indicated amounts of a TNF-blocking antibody (αTNF Ab) or an isotype-matched control antibody (Control Ab) (**b**), or 1 μg of fMA with or without recombinant TNF (rTNF) (**b**). NO production was determined by Griess assay. Data represent as the mean ± SEM of triplicate assays from three independent experiments. The statistical significance was calculated by one-way ANOVA followed by Bonferroni’s test. **p < 0.01, ***p < 0.001, n.s. not significant. **c** Representative images of the hematoxylin and eosin-stained lungs from WT, *Clec4e*^*−/−*^, or *Card9*^*−/−*^ mice at day 7 after intravenous injection of 50 μg TDM or 250 μg fMA emulsion. The images are representative of two independent experiments. Scale bars: 0.1 mm. **d**–**f** Peritoneal lavages were collected at 4, 24, and 72 h after intraperitoneally injection of 500 μg fMA or 100 μg TDM emulsion to WT mice (*n* = 4). MCP-1 and TNF concentrations in the lavages were measured by ELISA, and *Nos2* mRNA levels were measured by quantitative RT-PCR. **e** Flow cytometric analysis of peritoneal neutrophils (CD11b^+^Ly6G^+^F4/80^−^) and monocyte-derived macrophages (CD11b^+^Ly6G^−^F4/80^low^ SPMs) from WT mice (*n* = 4) at 48 and 72 h post injection of MA or TDM emulsions as in **d**. **f**, **g** WT or *Trem2*^*−/−*^ mice were injected intraperitoneally with fMA or control (vehicle) emulsion. The concentrations of MCP-1 (control, *n* = 4; fMA, *n* = 6; at 4 h) and TNF (*n* = 4; at 72 h) in the peritoneal lavages was measured by ELISA (**f**). Peritoneal exudate cells (*n* = 4; at 72 h) was analyzed as in **e** and **g**. **h**–**k** WT mice w**e**re injected intraperitoneally with TDM, fMA, or control (vehicle) emulsion, and infiltrated macrophages at days 1, 2, and 3 were analyzed by flow cytometry for the expression of CD38 and iNOS (**h**). The numbers of total recruited monocyte-derived macrophages (CD11b^+^Ly6C^+^F4/80^+^) (**i**), the M1 macrophages (CD11b^+^Ly6C^+^F4/80^+^CD38^high^NOS^+^) (**j**), and permissive macrophages (CD11b^+^Ly6C^+^F4/80^+^CD38^dull^NOS^−^) (**k**) at day 3 are shown. Data in **d**–**k** represent as the mean ± SEM from at least three independent experiments. The statistical significance was calculated by two-way ANOVA followed by Bonferroni’s test (**f**–**g**) or by two-tailed unpaired *t* test (**i**–**k**). *p < 0.05, **p < 0.01, ***p < 0.001, n.s. not significant. Source data are provided as a Source data file.

Given that NO plays a pivotal role in controlling mycobacterial infections^59,60^, we investigated the action of TREM2 and Mincle on NO production by macrophages. We observed that TDM stimulation strongly induced NO production by BMDMs, as reported previously^30^, while fMA did not substantially induce NO production (Fig. 5a). This was consistent with the observed increase in *Nos2* expression, which encodes iNOS, after stimulation with TDM but not fMA (Supplementary Fig. 5a). In addition, the TDM-induced NO production by macrophages was inhibited following the addition of a TNF-blocking antibody (Fig. 5b). Inversely, addition of recombinant TNF to the fMA-stimulated culture induced NO production by macrophages (Fig. 5b), indicating that NO production was dependent on TNF, which is consistent with previous findings^61–63^.

We then characterize the inflammation triggered via Mincle and TREM2 in vivo. Because TDM induces lung granulomas in mice via the Mincle/FcRγ pathway^30^, we examined whether fMA had a similar effect. Intravenous injection of a TDM (oil-in-water) emulsion into mice induced massive granulomatous lesions in the lungs, which was completely abolished in *Clec4e*^*−/−*^ mice (Fig. 5c), as reported previously^30^. In addition, this effect was also absent in *Card9*^*−/−*^ mice, indicating that TDM-induced granuloma formation was dependent on Mincle/CARD9 signaling. By contrast, we did not observe lung granulomatous lesions in mice injected with an fMA emulsion (Fig. 5c), suggesting that fMA cannot activate CARD9 signaling. To further explore the innate immune response induced by TDM or MA in vivo, we intraperitoneally injected the TDM or fMA emulsion into mice and measured the level of MCP-1 and TNF, and *Nos2* mRNA levels, as well as the number of recruited inflammatory cells, induced in the peritoneal cavities. We detected MCP-1 in peritoneal lavages following respective fMA and TDM administrations at similar levels, with peak MCP-1 levels observed at 4 and 24 h post injection, respectively (Fig. 5d). However, TNF production and *Nos2* mRNA expression were induced only by TDM but not fMA (Fig. 5d), which was consistent with results from the in vitro-stimulated macrophages (Fig. 4). Moreover, we observed that TDM recruited substantial numbers of macrophages [CD11b^+^Ly6G^−^F4/80^low^ small peritoneal macrophages (SPMs)], Supplementary Fig. 5b) and neutrophils (CD11b^+^Ly6G^+^F4/80^−^, Supplementary Fig. 4b) to the cavity, whereas fMA recruited comparable numbers of macrophages as TDM, but fewer neutrophils only immediately after administration (Fig. 5e). Importantly, MCP-1 level (Fig. 5f) and the number of recruited cells (Fig. 5g) induced by fMA in *Trem2*^*−/−*^ mice were almost the same as those induced by the control vehicle administration, indicating that the fMA-induced inflammation was dependent on TREM2.

To investigate the phenotype of TDM- or fMA-induced macrophages, we examined the expression of the inflammatory M1-macrophage markers iNOS and CD38 (ref. ^64^) in the recruited macrophages (Fig. 5h and Supplementary Fig. 5c). Intraperitoneal administration of TDM and fMA recruited comparable numbers of macrophages to the cavity (Fig. 5i); however, although TDM-induced macrophages exhibited a CD38^high^iNOS^+^ phenotype (Fig. 5j), those induced by fMA had reduced CD38 expression and were negative for iNOS (Fig. 5k). This phenotype resembles a previously reported phenotype associated with permissive macrophages that are recruited by a subset of pathogenic mycobacteria expressing the virulence lipids PDIM and PGL in an MCP-1- and CCR2-dependent manner, and provide a niche for mycobacterial propagation^21,65^.

Collectively, these results suggested that triggering of the Mincle–CARD9 pathway elicited lung granuloma formation and recruited M1-type mycobactericidal macrophages producing TNF and NO, whereas TREM2 activation recruited mycobacterium-permissive macrophages lacking TNF and NO production.

### TREM2 deficiency exacerbates Mincle-induced inflammation

**Fig. 6:**
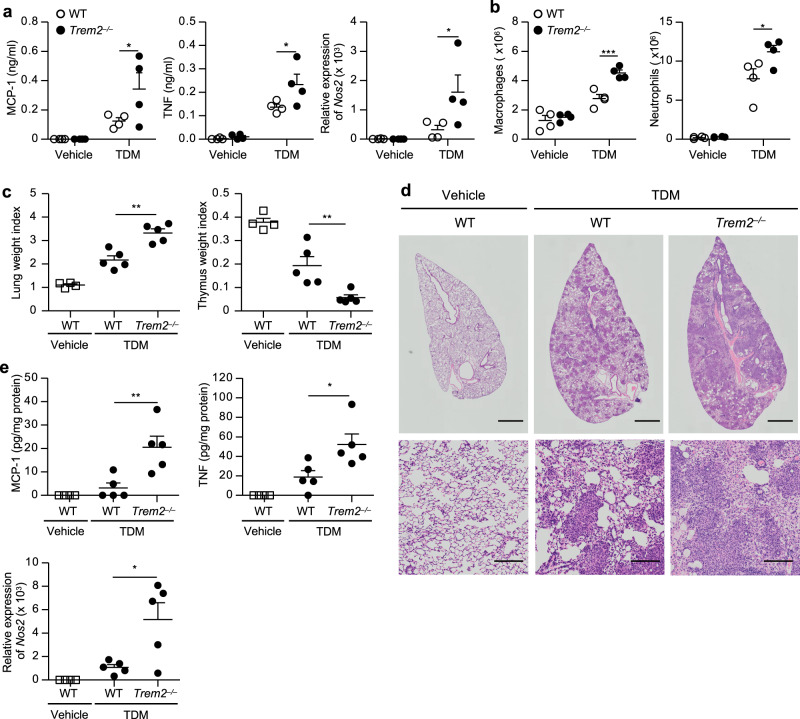
TREM2 deficiency exacerbates Mincle-induced inflammation. **a**, **b** WT or *Trem2*^*−/−*^ mice (*n* = 4) were intraperitoneally injected with 100 μg TDM or control (vehicle) emulsion. TNF and MCP-1 in the peritoneal lavages were measured by ELISA and *Nos2* mRNA levels in the peritoneal cells were measured by qRT-PCR at 24 h post injection (**a**). The numbers of macrophages and neutrophils in the peritoneal cavities at 72 h post injection were analyzed by flow cytometry (**b**). **c**–**e** WT or *Trem2*^*−/−*^ mice (*n* = 5) were intravenously injected with 50 μg TDM or control emulsion, and the lungs and thymuses were collected at day 7 after the injection. LWI and TWI are shown (**c**). Representative hematoxylin and eosin-stained sections of lung lobes from WT and *Trem2*^*−/−*^ mice (scale bars: upper panels, 1 mm; lower panels, 0.1 mm) are shown (**d**). Cytokine concentration in lung homogenates was measured by ELISA, and *Nos2* mRNA levels in the lungs were measured by qRT-PCR (**e**). Data in **a**–**c** and **e** are presented as mean ± SEM and are representative of at least two independent experiments. The statistical significance was calculated by two-way ANOVA followed by Bonferroni’s test (**a**, **b**) or by two-tailed unpaired *t* test (**c**, **e**). *p < 0.05, **p < 0.01, ***p < 0.001. Source data are provided as a Source data file.

To investigate the relevance of TREM2 inhibition of Mincle-induced macrophage activation in vivo, we assessed the impact of TREM2 deficiency on tissue inflammation induced by TDM administration. Intraperitoneal injection of TDM emulsion into *Trem2*^*−/−*^ mice resulted in significantly higher levels of TNF and MCP-1 production and increased *Nos2* mRNA expression (Fig. 6a), as well as higher numbers of macrophages and neutrophils to the peritoneal cavities (Fig. 6b), as compared to those observed in WT mice. Intravenous injection of the TDM emulsion into mice induces lung swelling (increased lung weight index: LWI) and thymic atrophy (decreased thymic weight index: TWI) dependent on the Mincle–FcRγ pathway^30^. We observed that *Trem2*^*−/−*^ mice displayed significantly higher LWI and lower TWI than WT mice following TDM injection (Fig. 6c). Histopathological analysis of the lungs revealed that the granulomatous lesions were markedly more prominent in *Trem2*^*−/−*^ mice (Fig. 6d). In addition, specific pathological features in the form of prominent vasculitis and edema, indicating accelerated inflammation, were observed in the lungs of *Trem2*^*−/−*^ but not WT mice (Supplementary Fig. 6). Consistent with these results, the levels of TNF and MCP-1 production and *Nos2* mRNA expression in the inflamed lungs were significantly higher in *Trem2*^*−/−*^ mice than in WT mice (Fig. 6e). These results suggested that TREM2 suppressed Mincle-induced inflammation in vivo.

### TREM2 deficiency accelerates the clearance of mycobacterial infection

**Fig. 7:**
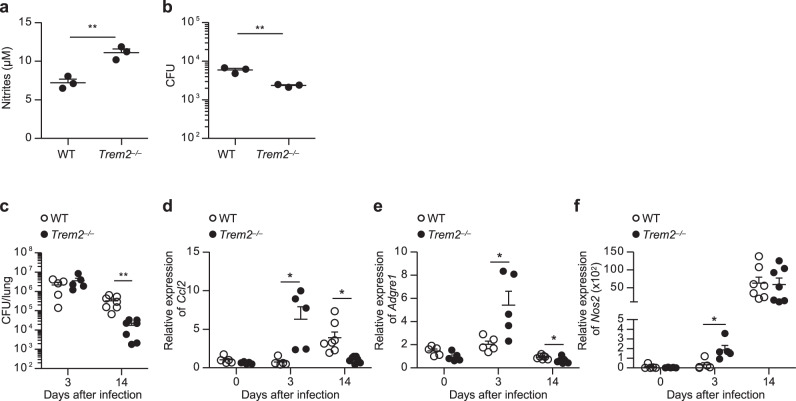
TREM2 deficiency accelerates the clearance of mycobacterial infection. **a**, **b** WT or *Trem2*^*−/−*^ BMDMs were infected in vitro with *M. bovis* BCG (MOI 10) for 4 h. After washing, the cells were further cultured for 24 h, and nitrite concentration in the culture supernatant was measured by Griess assay (**a**). The cells were collected, lysed, and CFUs of *M. bovis* BCG were counted (**b**). The statistical significance was calculated by two-tailed unpaired *t* test. **c**–**f** WT or *Trem2* ^*−/−*^ mice (day 3, *n* = 5; day 14, *n* = 7) were infected intratracheally with 7.5 × 10^6^ CFU of *M. bovis* BCG. The lung tissues were collected at days 3 and 14 after infection, homogenized, and CFU were determined (**c**). The expressions of *Ccl2*, *Adgre1*, and *Nos2* in the lungs from uninfected control (*n* = 5) and infected mice were analyzed by qRT-PCR (**d**–**f**). Data are presented as the mean ± SEM and are representative of two independent experiments. The statistical significance was calculated by two-tailed unpaired *t* test. *p < 0.05, **p < 0.01. Source data are provided as a Source data file.

Since our data suggested a suppressive role for TREM2 in the microbicidal innate immune response via Mincle, we investigated the impact of TREM2 deficiency on the clearance of mycobacterial infection. First, we infected WT and *Trem2*^*−/−*^ BMDMs with *M. bovis* BCG in vitro, and assessed NO production and mycobacterial killing in the macrophages. NO production in *Trem2*^*−/−*^ BMDMs was significantly higher than in WT BMDMs (Fig. 7a). Accordingly, the number of intracellular BCG colony-forming units (CFUs) was significantly lower in *Trem2*^*−/−*^ BMDMs than in WT BMDMs (Fig. 7b). TREM2 deficiency did snot affect the phagocytic activity of BMDMs against *M. bovis* BCG (Supplementary Fig. 7), suggesting that the lower bacterial CFUs in *Trem2*^*−/−*^ BMDMs was likely resulted from the enhanced bactericidal activity, but not from an impaired phagocytosis.

Next, to investigate the relevance of this observation in vivo, we have intratracheally infected WT and *Trem2*^*−/−*^ mice with *M. bovis* BCG, and examined the bacterial burden and inflammatory mediators in the infected lungs at days 3 and 14 after infection. The bacterial burden in the lungs at day 14, but not day 3, was significantly lower in *Trem2*^*−/−*^ mice than in WT mice (Fig. 7c). Analyses of the cytokine production (MCP-1 and TNF), macrophage infiltration (estimated by *Adgre1* (F4/80) expression), and *Nos2* (iNOS) induction in the infected lungs detected a higher expression of *Ccl2* (MCP-1; Fig. 7d), *Adgre1* (Fig. 7e), and *Nos2* (Fig. 7f) in *Trem2*^*−/−*^ mice than in WT mice at day 3 after infection. TNF upregulation detected neither in the lungs of WT nor *Trem2*^*−/−*^ mice in any time points (Supplementary Fig. 8a). While in the day 14 lungs, the expression of *Ccl2* and *Adgre1* was rather lower in *Trem2*^*−/−*^ mice than in WT mice though *Nos2* expression was comparable, which was assumed to reflect the subsided inflammation by the accelerated bacterial clearance in the lungs of *Trem2*^*−/−*^ mice at this time point. Similarly, a higher inflammatory response at early time point and a lower bacterial CFUs at later time points in *Trem2*^*−/−*^ mice than in WT mice were observed, as well in an intraperitoneal *M. bovis* BCG infection (Supplementary Fig. 8b–e). To examine the impact of TREM2 deficiency on T-cell immunity against mycobacteria, we collected mediastinal lymph node cells from WT and *Trem2*^*−/−*^ mice at day 14 after the intratracheal infection, and stimulated them with the mycobacterial antigen purified protein derivative (PPD) to analyze their production of interferon (IFN)-γ and IL-17A. We observed that TREM2 deficiency did not significantly affected the production of these cytokines by the lymph node cells (Supplementary Fig. 8f), suggesting that the accelerated bacterial clearance in the *Trem2*^*−/−*^ lungs was likely resulted from the enhanced bactericidal responses by macrophages in the early stage of infection rather than from the subsequent T-cell-mediated immunity. This hypothesis might be supported by the data that neither the addition of MA (Supplementary Fig. 8g) nor TREM2 deficiency (Supplementary Fig. 8f, h) significantly affected T-cell recall responses in mice vaccinated with complete Freund’s adjuvant (CFA) that contains mycobacterial components. Collectively, TREM2 plays a significant role in regulating the early bactericidal innate immune responses during mycobacterial infection.

## Discussion

In the present study, we identified the DAP12-associated TREM2 as a receptor for mycobacteria by the screening using the ITAM-coupled receptor-Fc fusion protein library. TREM2 is expressed on various myeloid cells, including macrophages and microglia^66^, and *Trem2* deficiency is reportedly related to various neurodegenerative diseases. Homozygous loss-of-function mutations of *Trem2* or *Tyrobp* (*Dap12*) cause Nasu-Hakola disease accompanied by demyelination and axonal loss^67,68^. Single-nucleotide polymorphisms, including the TREM2 R47H variant, increase the risk of dementia, including frontotemporal lobar degeneration, Parkinson’s disease, Alzheimer’s disease, and amyotrophic lateral sclerosis^46–49^. These genetic associations highlight the physiological importance of TREM2 and its ligand recognition. Previous reports indicate that TREM2 binds to anionic ligands of bacteria^69^, lipo-oligosaccharide (LOS) of *Neisseria gonorrhoeae*^70,71^, several brain lipids associated with fibrillar amyloid β (ref. ^45^), and apolipoproteins^72^. Although these ligands have been identified, ligand structures prerequisites for TREM2 recognition remain unknown. Here, we identified MA-containing lipids as TREM2 ligands and found that their branched alkyl chains are required for TREM2 recognition via a structure–activity relationship analysis. The removal of branched alkyl chains from LOS by O-deacetylation diminishes TREM2–LOS interaction^71^, which implicates the lipid moiety of LOS in the interaction with TREM2. In addition, TREM2 ligands in the brain include phospholipids or sphingolipids carrying branched alkyl chains^45^; therefore, our findings agree with previous results and might offer insight into future searches for unknown TREM2 ligands.

We found that macrophage activation by glycosylated versus non-glycosylated MA lipids resulted in different patterns of response with different receptor requirements. While glycosylated MAs were recognized by Mincle, and induced the production of MCP-1, TNF, and NO, non-glycosylated MAs were recognized by TREM2 and induced only MCP-1 production. A previous report showed that human Mincle, but not mouse Mincle could recognize GroMM and induces TNF production in primary human macrophages^73^, suggesting that the range of structures of MA ligands recognized by Mincle might differ between humans and mice. Nevertheless, Hattori et al. showed in this report that the binding affinity of human Mincle to GroMM was ~100-fold lower than that to TDM, indicating the importance of the sugar moiety for the recognition by Mincle, regardless of species. Importantly, our data showed that mouse Mincle was also capable of binding to GroMM at higher concentrations, although it was not essential for macrophage response to GroMM. Future studies should clarify the contributions of TREM2 and Mincle to recognition of MA-containing lipids in humans.

MCP-1 plays a role in the early stages of Mtb infection^53,54^. In addition, higher levels of MCP-1 production are associated with greater severity of TB in human patients^74^. The polymorphism of at position −2518 the MCP-1 promoter is associated with TB susceptibility^75,76^, and the odds of developing TB is 2.3- to 5.5-fold higher in patients with MCP-1 genotypes AG and GG relative to those with the AA genotype. Patients carrying the AG or GG genotype harbor extremely high concentrations of MCP-1, which inhibit the expression of IL-12 (ref. ^76^), suggesting that elevated MCP-1 production and lower inflammatory cytokine levels promote TB development. This is a similar phenomenon to that observed following TREM2 recognition of non-glycosylated MAs in the present study. Mycobacterial cell wall components, PDIM and PGL, enhance infectivity by selective induction of MCP-1 for the recruitment of the permissive macrophages^77^. These findings indicate that exclusive production of MCP-1 via TREM2 is presumably beneficial to mycobacteria.

The recruitment of permissive macrophages and inhibition of Mincle–FcRγ–CARD9 signaling are key functions of TREM2. Here, we found that TREM2/DAP12 signaling specifically inhibited Mincle–FcRγ–CARD9 signaling, but not TLR signaling. However, previous reports show that TREM2 deficiency enhances cytokine production induced by TLRs^78,79^. Although the reason for this discrepancy is unknown, upregulated signaling via Mincle might be responsible for the enhanced TLR response. Because Mincle recognizes damaged cell-derived endogenous ligands^80,81^, Mincle expression induced by TLR stimulation^30^ might subsequently amplify the TLR response^82^ depending on cell condition. Alternatively, TREM2 binding to TDM (Fig. 3) but not TLR ligands could generate significant inhibitory signals upon TDM stimulation; therefore, TREM2 deficiency might result in more obvious effects on the signaling via Mincle than that via TLRs.

The composition of MA-containing lipids dynamically changes in response to the external environment. TDM is a major glycosylated MA in the mycobacterial cell wall in culture, but in the host under glucose-rich condition, TDM synthesis is downregulated, and GMM is produced by mycolytransferases using host-derived glucose^7^. Because glycosylated MAs exhibit potent adjuvant activity^83^, the presence of these lipids preferentially activates the immune response through Mincle to eliminate mycobacteria. Lipid composition differs in latent mycobacterial infections. GroMM might be associated with latent mycobacteria, given that GroMM-reactive T cells are observed only in latent but not active TB cases^15,73^. In addition, levels of glycosylated MAs decrease in Mtb in a nonreplicating dormant-like state^6^. Therefore, we speculate that the recognition of latent mycobacterial cell walls by TREM2 might lead to the recruitment of permissive macrophages, thereby promoting chronic infection (Supplementary Fig. 9).

Macrophages or microglia phagocytize bacteria or apoptotic cell debris through TREM2 (refs. ^70,84–86^). Macrophages are roughly classified into M1 and M2 subtypes that exert opposite effects on the inflammatory response^87^. M1 macrophages are pro-inflammatory, whereas M2 macrophages are anti-inflammatory and exhibit phagocytic activity to promote tissue repair and homeostasis. Unlike FcRγ-coupled mycobacterial receptors including Mincle, TREM2–DAP12 signaling seemingly confers M2-like anti-inflammatory properties on macrophages. Indeed, IL-4, which skews macrophage polarization toward M2, induces TREM2 expression in macrophages^79^. In addition, TREM2 deficiency impairs wound healing of colonic mucosal injures accompanied by diminished M2 differentiation and increased TNF and IFN-γ production^88^. Moreover, TREM2 plays an important role in maintaining homeostasis in the brain by clearance of dead cells or amyloid-β through brain-lipid recognition^84^. Therefore, mycobacteria presumably harness the functions of TREM2 by stimulating TREM2 with MA-containing lipids to gain niches suitable for their propagation.

Collectively, our data suggest that TREM2–DAP12 signal activation by non-glycosylated MAs, which are associated with virulent or latent mycobacterial infections, recruits permissive macrophages and suppress the mycobactericidal immune response induced by glycosylated MAs through Mincle–FcRγ–CARD9 signaling. Therefore, this study elucidated a mechanism by which mycobacteria control the host immune response by stimulating a host regulatory receptor with their specific cell wall lipids. Our findings suggest that targeting the TREM2–DAP12 pathway might represent a novel therapeutic intervention to control mycobacterial diseases.

## Methods

### Reagents

MAs from *M. bovis* BCG was prepared as described below. TDM (#T3034), LPS (#L3024), ABG (#10830), PA (#P0500), and HBA (#166898) were purchased from Sigma-Aldrich. Poly (I:C; #tlrl-picw), CpG-ODN (#tlrl-1585), Pam_3_CSK_4_ (#tlrl-pms) were purchased from InvivoGen. Oxidized (OX)-zymosan was kindly provided by Prof. Naohito Ohno (Tokyo University of Pharmacy and Life Sciences). MA from *R. equi* was kindly provided by Dr. Jun Miyazaki (Tsukuba University). LAM (#02449-61) was purchased from Nakalai tesque. THA (#T292210) was purchased from Wako pure chemical industries.

### Preparation of MA-containing lipids

Total lipids from mycobacteria were prepared as described previously^89^. Birefly, bacterial cells were extracted at 100 mg/ml in chloroform:methanol (C/M) (2:1, v/v) for 1 h at room temperature. After the centrifugation, the supernatant was filtered and evaporated in vacuo to weigh the recovered amounts of the total lipids. GMM and GroMM were prepared as described previously^7,16,90^. For GMM preparation, the total lipids dissolved in C/M (2:1) were added with 20 volumes of ice-cold acetone and incubated for 30 min on ice. After centrifugation, the pellet was washed with ice-cold acetone, dissolved with C/M (2:1), and fractionated by TLC (Analtech #Z26551) with chloroform/methanol/acetone/acetic acid (90:10:10:1, v/v), followed by fractionated with chloroform/acetone/methanol/water (50:60:2.5:0.6, v/v). The spot corresponding to GMM was scraped off the silica gel plate and extracted with C/M (2:1), dried, and rinsed several times with methanol at room temperature to remove residual contamination of glycopeptidolipids and phospholipids. For GroMM preparation, the total lipids extracted from the bacteria cultured in 7H10 medium containing 10% of glycerol were fractioned by two cycle of TLC with chloroform/ethyl acetone (5:1, v/v). The lipid spots were visualized with iodine vapor, and the spot corresponding to GroMM was scraped off the silica gel plate, followed by elution with C/M (2:1). fMAs were isolated from *M. bovis* BCG (Tokyo 172 strain, Japan BCG laboratory). The BCG cells were suspended in 85% tetrahydrofuran (THF)/water solution under a nitrogen atmosphere, followed by reflux with stirring for 1 h. The cell suspension was filtrated under pressure and washed with 75% THF/water solution. The residue was resuspended in 75% THF/water solution under a nitrogen atmosphere, followed by reflux with stirring for 1 h. The suspension was filtrated under pressure and washed with 75% THF/water solution three times and with methanol twice. Then, the bacterial cells were suspended in 50% 2-propanol/water solution containing 10% potassium hydroxide, followed by reflux with stirring for 2 h to complete alkaline hydrolysis of MA ester. After the refluxing, the suspension was cooled down on ice, and acidified with 6 M hydrochloric acid. The reaction mixture was extracted twice with *n*-heptane, and the *n*-heptane fraction was washed twice with water and then twice with 90% ethanol/water. Finally, the *n*-heptane fraction was concentrated in vacuo to obtain purified MAs. The final preparations of GMM, GroMM, and fMA were applied for TLC to confirm no extra no spots and for MADI-TOF mass spectrometry analyses to confirm the identity of the lipids.

### Mice

All mice used in this study were between the ages of 8–15 weeks. WT C57BL/6 mice were purchased from Kyudo co. *Trem2*^*−/−*^, *Card9*^*−/−*^, *Clec4e*^*−/−*^, *Tyrobp*^*−/−*^, and *Fcer1g*^*−/−*^ mice were generated previously^27,79,91–93^. All knockout mice were C57BL/6 genetic background. *Clec4e*^*−/−*^, *Tyrobp*^*−/−*^, and *Fcer1g*^*−/−*^ mice were kindly provided by Prof. Shizuo Akira (Osaka University), Prof. Toshiyuki Takai (Tohoku University), and Dr. Takashi Saito (RIKEN), respectively. All mice were maintained at the animal facility on Institute of Laboratory Animal Science Research Support Center Kagoshima University in a specific pathogen-free conditions room with a 12-h light/dark cycle at 22 ± 1 °C and 50 ± 10% humidity. All experiments using mice were approved by the Institutional Animal Research Committee of Kagoshima University and animals were treated in accordance with the ethical guidelines of Kagoshima University. Both male and female mice were used in this study but sex-matched mice were used for the comparison between groups in the same experiment.

### ITAM-coupled receptor-Fc preparation

Human IgG_1_ Fc region was integrated into *Acc*I site of pDisplay (Invitrogen #V660-20). The ectodomain of Clec2, Mincle, Clec9a, MGL-1, SIGNR1, SIGNR3, DCAR, Clec5a, DC-SIGN (human), TREM1, TREM2, TREM3, LMIR2, LMIR4, LMIR5, LMIR7, LMIR7, and LMIR8 (all from mouse origin except for DC-SIGN) were PCR amplified with the primers in Supplementary Table 1 using cDNA clones (purchased from DNAFORM) as templates, and were inserted into the upstream of the human IgG_1_ Fc region in-frame in pDisplay. pDisplay-mouse Dectin-1-Fc (kindly provided by Prof. Naohito Ohno, Tokyo University of Pharmacy and Life Sciences) and pME18S-mouse Dectin-2-Fc^80^ were used for the following Dectin-1-Fc and Dectin-2-fc expression, respectively. The plasmids were transfected into Freestyle 293 cells (Thermo Fisher Scientific #R79007) using Freestyle MAX reagent (Invitrogen #16447-100), and then cultured according to the manufacturer’s instruction. The culture supernatants were collected and then concentrated ~20 times by using VIVASPIN 20 MWCO 30 kDa (Sartorius Stedim Biotech #VS2022). These concentrated supernatants were used in the screening for proteins binding to mycobacteria. For the plate-coated lipid binding assay, TREM2- and Mincle-Fc fusion proteins were purified by using protein G columns, as descried previously^80^.

### Fractionation of mycobacteria

Ten mg of freeze-dried heat-killed Mtb H37Ra (Difco) was extracted with 500 μl of chloroform/methanol (2:1, v/v). After collecting the supernatant (sup fraction) by centrifugation, the de-lipidated precipitate (ppt fraction) was dried and resuspended with 500 μl (the same volume as the original extract) of PBS. While, water was added to the sup fraction so that the chloroform/methanol/water ratio was 8:4:3 (v/v), and further fractionated into the lipid-soluble (C:M) and the water-soluble (M:W) fractions. The C:M and M:W fractions were filled-up to 500 μl with chloroform/methanol (2:1, v/v) and methanol, respectively. Nontreated Mtb H37Ra was also dissolved in 500 μl of PBS. For the reporter assay, the nontreat and ppt fractions were serially diluted by four times with PBS and the same volume of these suspensions (μl/well) was directly added to the reporter cell culture in 96-well plates. The C:M and M:W fractions were serially diluted by four times with isopropanol, and the same volume of these solutions was added to the wells of 96-well plates and coated on the bottoms by solvent evaporation in a hood, followed by adding the reporter cells. The reporter cell assay was performed as below.

### 2B4-NFAT-GFP reporter cells

For the construction of DAP12 expression plasmid, SLAM signal sequence-FLAG-mouse DAP12 cassette was PCR amplified from pMX-IRES-GFP-FLAG-DAP12 (kindly provided by Dr. Takashi Saito, RIKEN) with the following primers: 5′-ACGCGTCGACGCCGCCACCATGGATCCCAAAGGATCCCT-3′ and 5′-AAGGAAAAAAGCGGCCGCCAGTGTGATGGA-3′. The amplified DNA was then digested with SalI and NotI, and cloned into XhoI and NotI sites of pMX-IRES-rCD2 to obtain pMX-IRES-rCD2-FLAG-DAP12. For the construction of TREM1 and TREM2 expression vectors, the coding sequences of mouse TREM1 (amino acids 21–230) and mouse TREM2 (amino acids 19–227) without signal sequences were PCR amplified with the primers: 5′-TACCCATACGATGTTCCAGATTACGCTGCCATTGTTCTAGAG-3′ and 5′-AAGGAAAAAAGCCGCTCATCCAAATGTCCT-3′; 5′-TACCCATACGATGTTCCAGATTACGCTCTCAACACCACGGTG-3′; and 5′-AAGGAAAAAAGCGGCCGCTCACGTACCTCCGGG-3′, respectively. SLAM signal sequence fused with HA was amplified by PCR using the primers: 5′-GAAGATCTGCCGCCACCATGAAAGACGACTTT-3′ and 5′-AGCGTAATCTGGAACATCGTATGGGTACACACCTCCACCTGT-3′ from pMX-IRES-rCD2-FLAG-DAP12. Then, the SLAM signal sequence-HA cassette was fused to TREM1 or TREM2 PCR products by subsequent PCR amplification, and cloned into pMX-IRES-hCD8 to obtain pMX-IRES-hCD8-HA-TREM1 and pMX-IRES-hCD8-HA-TREM2. pMX plamids were retrovirally transfected into 2B4-NFAT-GFP reporter cells (kindly provided by Dr. Takashi Saito, RIKEN), as described previously^80,94^. Breifly, pMX-IRES-rCD2-FLAG-DAP12 was transfected into a retroviral packaging cell line, Phoenix (kindly provided by Dr. Takashi Saito, RIKEN), with Lipofectamine LTX and Plus Reagent (Invitrogen #15338-100). The culture supernatants of Phoenix were collected after 24, 48, and 72 h, pooled, and centrifuged overnight 8000 × *g* to concentrate the virus. The concentrated viruses were added to 2B4-NFAT-GFP cells with 8 μg/ml polybrene (Nacalai tesque #12996-81) and centrifuged at 780 × *g* for 1 h for infection. The transfected cells were stained with PE-conjugated anti-rat CD2 antibody (Biolegend #210315) and anti-PE Microbeads (Miltenyi Biotec #130-048-801), and then purified by MACS (Miltenyi Biotec #130-042-201). The purifed 2B4-NFAT-GFP cells expressing DAP12 were cloned by serial dilution. Then, pMX-IRES-hCD8-HA-TREM1 or pMX-IRES-hCD8-HA-TREM2 were retrovirally transfected in the same way into 2B4-NFAT-GFP cells expressing DAP12 to produce 2B4-NFAT-GFP expressing DAP12 and TREM1 or TREM2, respectively. The transfected cells were stained with APC-conjugated antihuman CD8 antibody (Biolegend #300911) and anti-APC Microbeads (Miltenyi Biotec #130-090-855), and then purified by MACS. The purified cells were cloned by serial dilution. The R47H mutation in TREM2 was generated by KOD-Plus-Mutagenesis Kit (TOYOBO #SMK-101) using pMX-IRES-hCD8-HA-TREM2 as a template. 2B4-NFAT-GFP cells expressing FcRγ and Mincle were generated previously as described^80^. For the reporter assay, 5 × 10^4^ reporter cells in 96-well plate were incubated with mycobacteria, soluble PAMPs, or plate-coated lipids for 24 h and then data were collected by Cytoflex flow cytometer (BECKMAN COULTER) using CytExpert software version 2.3. Flow cytometry data were analyzed using FlowJo software version 10.5.3 (BD). Percent increase was calculated by dividing GFP MFI values after stimulation by those before stimulation.

### Screening for ITAM-coupled receptors binding to mycobacteria

Heat-killed Mtb H37Rv, Mtb H37Ra (Difco #231141), or *M. bovis* BCG were incubated with 20 μg/ml of ITAM-coupled receptor-Fc fusion proteins or control Fc fragment protein in RPMI 1640 medium for 1 h on ice. After two times washing with the medium, the mycobacterial cells were incubated with fluorescein isothiocyanate (FITC)-conjugated antihuman IgG secondary antibody (Jackson ImmunoResearch #709-095-149) for 30 min on ice. After a wash, the fluorescence intensity was analyzed by flow cytometer as above. The receptors that showed higher fluorescence intensities than the control values were judged as positive for binding.

### Plate-coated lipid binding assay

The lipids (MA, TDM, TDB, PA, THA, GMM, GroMM, and HBA) were dissolved in chloroform at 1 mg/ml, and then diluted with isopropanol to the working concentrations. Lipid binding assay was performed as described previously^95^. Briefly, 0.5 μg/well of fMA, GroMM, GMM, and TDM were coated on ELISA plates, and then incubated with 0.24–1000 nM purified Mincle-Fc or TREM2-Fc proteins in TSM buffer (20 mM Tris–HCl, 150 mM NaCl, 1 mM CaCl_2_, 2 mM MgCl_2_, pH 7.0) for 2 h at room temperature. The plates were washed four times with 150 μl of TSM buffer, and then incubated with antihuman IgG HRP secondary antibodies (abcam #ab6759) for 1 h at room temperature. The binding was detected by colorimetric assay using TMB substrate (Sumitomo Bakelite #ML-1120T) and the absorbance at 450 nm was measured by VERSAMax microplate reader, using SoftMaxPro software (Molecular Devices).

### In vitro macrophage stimulation

Peritoneal macrophages were prepared as described previously^96^. Briefly, 2 ml of 4% thioglycollate (Difco #225640) solution was intraperitoneally injected into mice. Five days after the injection, peritoneal cells were collected by washing the peritoneum cavity with 5 ml of RPMI 1640 medium containing 10% FCS and 2-mercaptoethanol (RPMI-10). The collected cells were cultured overnight in RPMI-10 and then the adherent cells were used for assays. BMDMs were prepared as described previously^27^. Briefly, bone marrow cells collected from WT, *Trem2*^−/−^, *Tyrobp*^−/−^, or *Clex4e*^−/−^ mice were cultured in RPMI-10 in the presence of 25 ng of recombinant murine M-CSF (PeproTech #AF-315-02) for 3 days, and then the adherent cells were collected as BMDMs. For in vitro cell stimulation, the lipid solutions were added into the 96-well flat bottom plates at 20 μl/well and then the solvent was completely evaporated in a hood before plating macrophages^30^. A total of 1 × 10^5^ cells/well were stimulated with plate-coated lipids or TLR ligands in RPMI-10. The culture supernatants after 24-h culture were collected and the concentrations of TNF (eBioscience #88-7324-88), IL-6 (eBioscience #88-7024-88), IL-12p40 (Biolegend #431604), IL-10 (Biolegend #431411), and MCP-1 (Biolegend #432701) were analyzed by ELISA kits, according to manufacturer’s instructions. For Syk inhibition, cells were incubated with 1 μM BAY-613606 (Calbiochem #574714) for 30 min prior to the stimulation.

### Measurement of NO production

BMDMs were stimulated in RPMI-10 with 0.1 or 1.0 μg per plate of plate-coated MA or TDM in the presence of 10 ng/ml of recombinant IFN-γ for 24 h. For the assessment of TNF involvement in NO production, carrier-free recombinant mouse TNF (Biolegend #575202), LEAF purified anti-mouse TNF-α antibody (MP6-XT22; BioLegend #506331), or LEAF purified Rat IgG_1_ κ Isotype Ctrl antibody (BioLegend #400457) were added to the culture. The culture supernatants were collected and mixed with Griess reagent (1% sulfanilamide, 0.1% *N*-(1-naphthyl) ethylenediamine dihydrochloride, and 2.5% phosphoric acid) at a 1:1 ratio, reacted for 10 min, and the absorbance at 550 nm was measured by a VERSAMax microplate reader using SoftMaxPro software. The nitrite concentration was calculated according to the standard curve.

### Quantitative real-time PCR

Total RNA was isolated from cells using Sepasol-RNA I Super G RNA-isolation kit (Nacalai Tesque #09379). After the removal of DNA contamination by DNase I (Nippon Gene #312-05951), the total RNA was reverse-transcribed with ReverTra Ace qPCR RT Master Mix (TOYOBO #FSQ-201) to synthesize cDNA. Quantitative real-time PCR (qRT-PCR) was performed with THUNDERBIRD SYBR qPCR Mix (TOYOBO #QPS-201), using StepOnePlus and StepOne Software version 2.3 (Thermo fisher scientific). Sequences of the specific primer sets used in this PCR were listed in Supplementary Table 2. The relative expression levels of the genes were calculated by ΔΔCt method normalized by *Gapdh*.

### MA and TDM emulsions for i.p. administration

A total of 10 mg MA or 2 mg TDM was dissolved in 1 ml of Bayol F (SERVA Electrophoresis #14500.01) at 64 °C, then mixed with 1 ml of PBS using a handy homogenizer (RELIEF). A total of 100 μl of the emulsion was intraperitoneally injected into WT or *Trem2*^*−/−*^ mice. The emulsion without lipids was injected as a vehicle control. Peritoneal lavages were collected by washing the peritoneum cavity with RPMI-10. Concentration of cytokine in the lavage was measured by ELISA kit, as described above.

### TDM emulsion for i.v. administration

TDM emulsion was prepared as described previously^30^. Briefly, 1 mg of TDM was dissolved in 180 μl of Bayol F at 64 °C, then mix with 1.8 ml of 1.1% Tween 80/0.9% NaCl solution to make emulsion. A total of 100 μl of the emulsion including 50 μg of TDM was intravenously injected into WT and *Trem2*^*−/−*^ mice. At day 7, the thymuses, lungs, and body weights were measured and the thymus or lung weights were divided by body weights to calculate lung or thymus weight index, respectively. The left lobes were then fixed in 4% paraformaldehyde, and embedded in paraffin and stained with hematoxylin and eosin solution. The slides were analyzed by All-in-One fluorescence microscope BZ-X700 using BZ-H4XD software (KEYENCE). The rest of lung tissues were homogenized by gentleMACS (Miltenyi Biotec), running program lung_01, followed by incubation for 35 min at 37 °C in PBS containing 0.2 mg/ml Liberase Tm (Roche #5401119001) and 25 μg/ml DNase I (Roche #11 184 932 001), then program lung_02, as described previously^97^, and the concentration of cytokines in homogenates were measured by ELISA kit, as described above.

### Flow cytometry

For the analysis of infiltrated neutrophils and macrophages, cells in the peritoneal lavage were stained with FITC-conjugated anti-CD11b antibody (M1/70, eBioscience #11-0112-41), PE-conjugated anti-Ly6G antibody (1A8, eBioscience #12-9668-82), and APC-conjugated anti-F4/80 antibody (BM8.1, TONBO biosciences #20-4801) after the Fc-blocking with anti-CD16/CD32 antibody (2.4G2, TONBO biosciences #70-0161). For the intracellular iNOS (NOS2) staining, the cells after the Fc-blocking were stained with PE-Cy7-conjugated anti-Ly6C (HK1.4, Biolegend #128017), PE-conjugated anti-F4/80 (BM8.1, Biolegend #123109), and FITC-conjugated anti-CD38 (90, Biolegend #102705), and then fixed and permeabilized with BD Cytofix/Cytoperm kits (BD Biosciences #BDB554714). After washing, the cells were stained with APC-conjugated anti-NOS2 antibody (CXNFT, eBioscience #17-5920-80). The stained cells were analyzed using CytoFLEX flow cytometer (Beckman Coulter), using CytExpert software version 2.3 and Flowjo software version 10.5.3 (BD).

### Immunization of mice

Immunization of mice was performed as described previously^98^. Briefly, ovalbumin (200 μg/ml; OVA, Sigma-Aldrich #A5503) dissolved in PBS was emulsified in equal volume of Freund’s incomplete adjuvant (IFA, Chemicon #AR002), or IFA containing either 10 mg/ml of Mtb H37Ra (CFA) or MA, or both. Mice were intradermally injected at the tail base with 100 μl of these emulsions. Twenty-eight days after immunization, spleen cells were collected and stimulated ex vivo with 50 μg/ml of OVA for 3 days in RPMI-10 (1.0 × 10^6^ cells/ml). The concentration of IFN-γ and IL-17A in the culture supernatants were measured by ELISA (Biolegend #430801 and R&D systems #DY5390, respectively).

### Phagocytic assay

*M. bovis* BCG (Tokyo 172 strain, Japan BCG laboratory #876391) was labeled with FITC (SIGMA #F7250), as described previously^99^. Briefly, 6.0 × 10^7^ CFU of *M. bovis* BCG were resolved in 200 μl of 0.1 M carbonate–bicarbonate buffer (pH 9.6)/0.05% Tween 80 containing of 30 μg/ml FITC, and incubated at 37 °C for 15 min. The FITC-labeled mycobacteria were washed with PBS/0.05% Tween 80. For phagocytosis assay, WT or *Trem2*^*−/−*^ BMDMs (1 × 10^5^) were incubated with 1 × 10^6^ (MOI 10:1) of FITC-labeled mycobacteria in a 96-well plate for 4 h at 37 °C. Negative controls were prepared in identical conditions but incubated on ice (0 °C). The phagocytosis was stopped by washing with ice-cold PBS. The phagocytosed mycobacteria were analyzed by flow cytometer after addition of 0.02% Trypan Blue to quench FITC fluorescence of extracellular but not internalized mycobacteria^100^.

### *M. bovis* BCG infection

In vitro infection of *M. bovis* BCG was performed as described previously^101^. Briefly, BMDMs (1 × 10^5^) were incubated in a 96-well plate with 1 × 10^6^ (MOI 10:1) of live *M. bovis* BCG for 4 h at 37 °C. After the infection, uninfected bacteria were washed-out with RPMI-10 and the infected cells were further cultured for 24 h in RPMI-10. The supernatant was collected and he nitrite concentration was measured by Griess assay. For CFU determination, the cells were disrupted by water and plated on Middlebrook 7H10 agar (Difco #262710) and number of BCG colonies was counted after 3-week culture.

Lung *M. bovis* BCG infection was performed as described previously^102^. Briefly, mice were intratracheally inoculated with 7.5 × 10^6^ CFU of *M. bovis* BCG. The lung tissues were collected at days 3 and 14 after infection, and homogenized by gentleMACS. The homogenates were serially diluted in water and plated on Middlebrook 7H10 agars and colonies were counted, as described above. Total RNA was extracted from the lung homogenates and gene expression was analyzed by qRT-PCR, as described above.

For T-cell recall response to mycobacterial antigens, mediastinal lymph node cells were collected at day 14 after intratracheal infection and stimulated with 5 μg/ml of tuberculin PPD (Japan BCG laboratory #114015) for 3 days. Concentration of IFN-γ and IL-17 in the culture were measured by ELISA

Intraperitoneal *M. bovis* BCG infection was performed, as described previously^103^. Briefly, WT and *Trem2*^*−/−*^ mice were infected intraperitoneally with 5 × 10^6^ CFU of *M. bovis* BCG. The peritoneal lavages were collected at 4 h, day 1, and day 3 after infection by washing the peritoneal cavity with 1 ml of RPMI-10. The concentration of cytokines in the lavage was analyzed by ELISA. The peritoneal cells were analyzed by flow cytometry. CFU was counted as described above.

### Statistical analysis

All statistical analyses were carried out using GraphPad Prism 5 software. Two-tailed unpaired *t* test was performed for the comparisons between two groups. One-way ANOVA or two-way ANOVA with Bonferroni post hoc test were performed for the comparison of multiple groups. *P* values <0.05 were considered statistically significant.

### Reporting summary

Further information on research design is available in the Nature Research Reporting Summary linked to this article.

## Supplementary information

Supplementary Information

Reporting Summary

## Data Availability

All data generated or analyzed during this study are available from the corresponding author upon reasonable request. Source data are provided with this paper.

## Source data

Source Data
