# Supplementary material for: TREM2 is a receptor for non-glycosylated mycolic acids of mycobacteria that limits anti-mycobacterial macrophage activation
Source: Nat Commun. 2021 Apr 16;12:2299. doi: 10.1038/s41467-021-22620-3 (PMC8052348; doi:10.1038/s41467-021-22620-3)
Supplement: Supplementary file 1 — Supplementary Information [file 41467_2021_22620_MOESM1_ESM.pdf]

Supplementary Information

Contrasting recognition and responses by macrophages via TREM2 versus Mincle to mycobacterial mycolic acid-containing lipids

Ei'ichi Iizasa[1], Yasushi Chuma[2], Takayuki Uematsu[3], Mio Kubota[4], Hiroaki Kawaguchi[5], Masayuki Umemura[6], Kenji Toyonaga[1,7], Hideyasu Kiyohara[2], Ikuya Yano[2,8], Marco Colonna[9], Masahiko Sugita[10], Goro Matsuzaki[6], Sho Yamasaki[7], Hiroki Yoshida[4] and Hiromitsu Hara[1*]

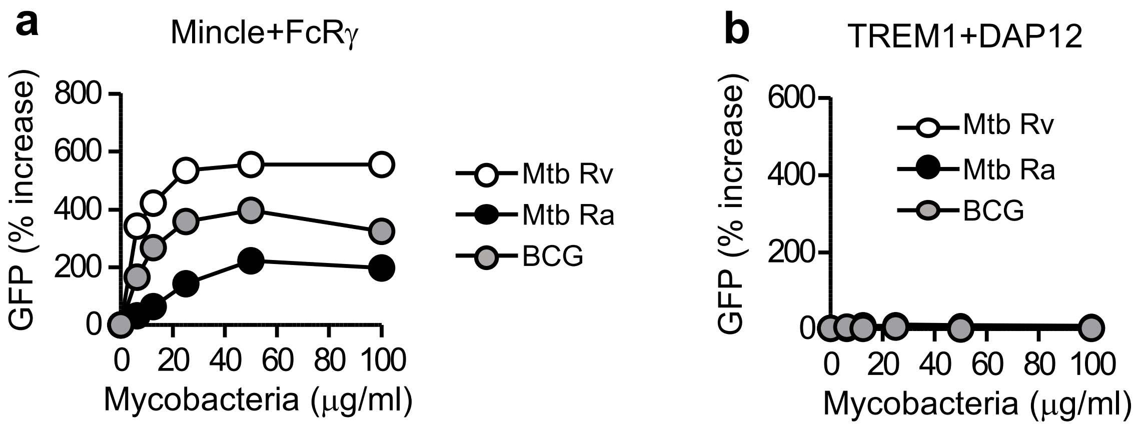

Supplementary Figure 1

Response of TREM1- or Mincle-reporter cells to mycobacteria. NFAT-GFP-reporter cells expressing Mincle+FcRγ (**a**) or TREM1+DAP12 (**b**) were stimulated with the indicated amounts of heat-killed *Mtb* H37Ra or H37Rv or *M. bovis* BCG for 24 h, and GFP fluorescence was analyzed by flow cytometry. Data are presented as the mean ± SEM of duplicate assays and are representative of three independent experiments. Source data are provided as a Source Data file.

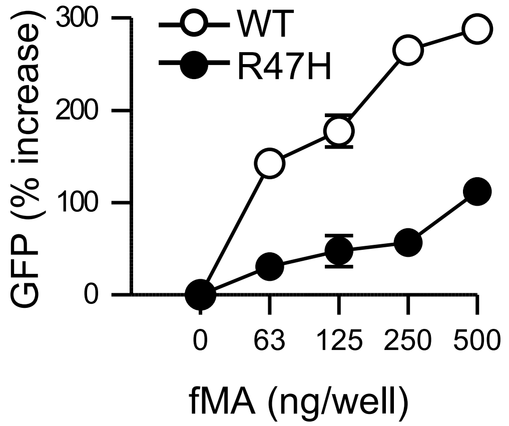

Supplementary Figure 2

The R47H mutation affects TREM2 recognition of MA. NFAT-GFP-reporter cells expressing WT or TREM2 R47H +DAP12 were stimulated with the indicated amounts of fMA coated on the plates for 24 h, and GFP fluorescence was measured by flow cytometry. Data are presented as the mean ± SEM of duplicate assays and are representative of three independent experiments. Source data are provided as a Source Data file.

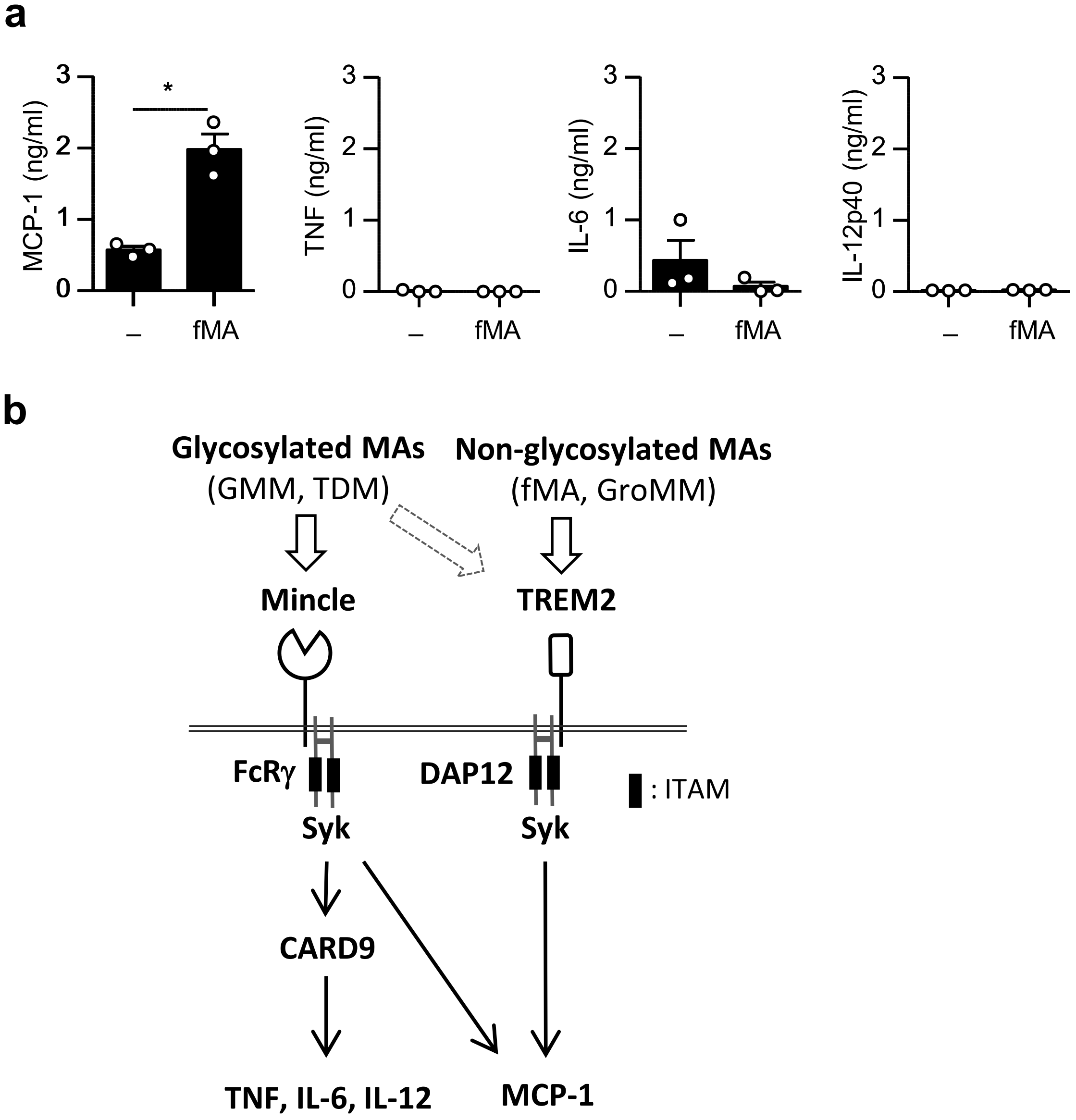

Supplementary Figure 3

Macrophages respond to non-glycosylated MAs in a TREM2/DAP12-dependent but CARD9-independent manner to produce MCP-1 but not inflammatory cytokines. (**a**) Peritoneal macrophages from C57BL/6 mice were left unstimulated (–) or stimulated with 1 μg/well of plate-coated fMA from *M. bovis* BCG for 24 h. MCP-1, TNF, IL-6, and IL-12p40 concentrations in the culture supernatants were measured by ELISA. Data are presented as the mean ± SEM of triplicate assays and are representative of three independent experiments. The statistical significance was calculated by two-tailed unpaired *t*-test. $*p < 0.05$ (**b**) Schematic model of TREM2, Mincle, and downstream ITAM signaling required for macrophage production of MCP-1 and inflammatory cytokines, such as TNF, induced by MA-containing lipids. Source data are provided as a Source Data file.

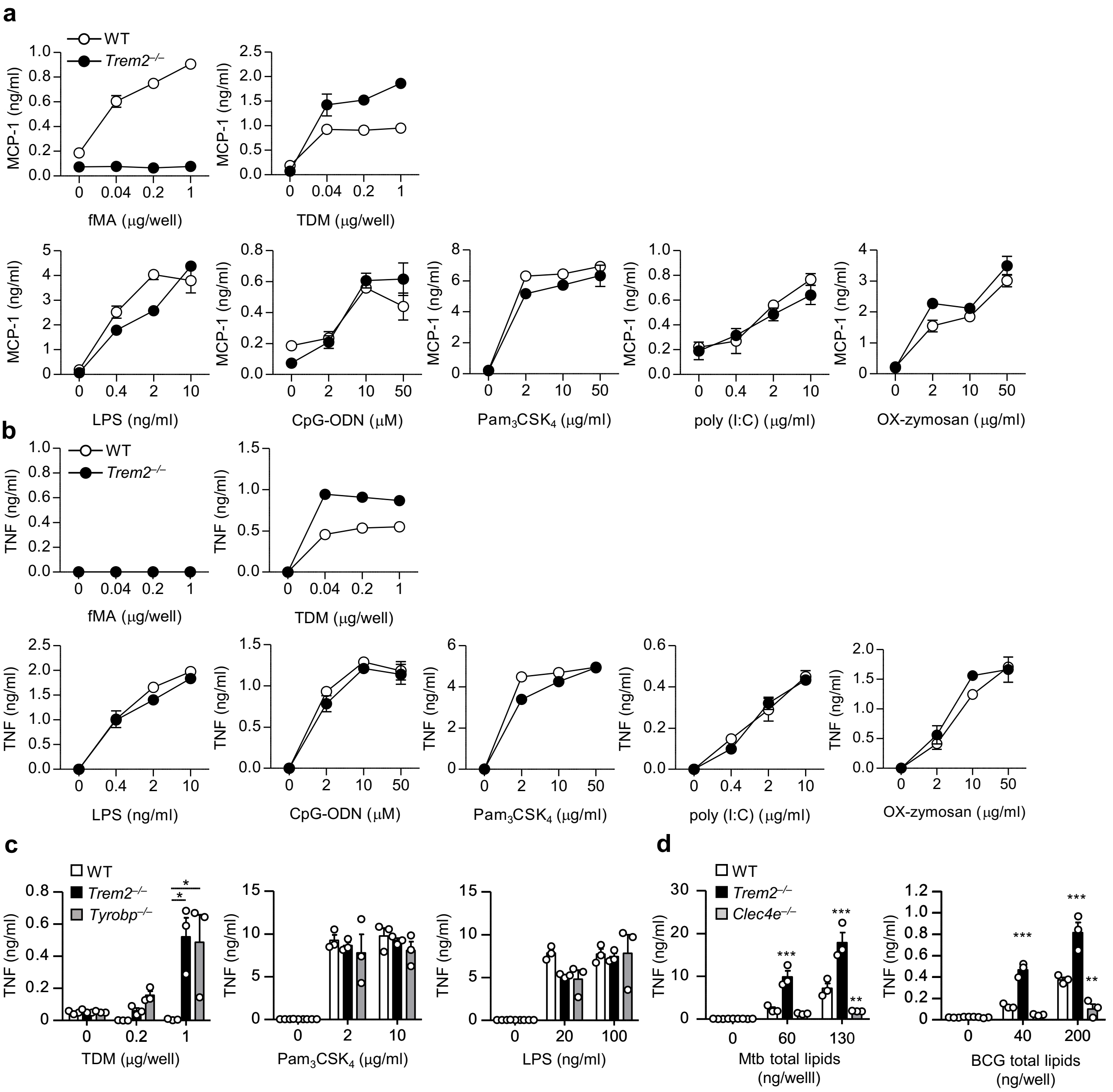

Supplementary Figure 4

TREM2 selectively inhibits macrophage activation induced by Mincle but not that induced by TLRs or Dectin-1. Peritoneal macrophages from WT and *Trem2*$^{-/-}$ mice (**a, b**) or BMDMs from WT, *Trem2*$^{-/-}$, *Tyrobp*$^{-/-}$, and *clec4e*$^{-/-}$ mice (**c, d**) were stimulated with the indicated amounts of fMA (for TREM2; **a, b**), TDM (for Mincle; **a–c**), Pam$_3$CSK$_4$ (for TLR2; **a–c**), LPS (for TLR4; **a–c**), Poly (I:C) (for TLR3; **a, b**), CpG-ODN (for TLR9; **a, b**), or oxidized (Ox)-zymosan (for Dectin-1; **a, b**), or stimulated with total lipids (**d**) extracted from *Mtb* H37Ra (Mtb) or *M. bovis* BCG (BCG) for 24 h, and the levels of MCP-1 (**a**) and TNF (**b-d**) in the culture supernatants were measured by ELISA. Data are presented as the mean ± SEM of triplicate assay and are representative of two independent experiments. The statistical significance was calculated by two-way ANOVA followed by Bonferroni's test. *$p < 0.05$, **$p < 0.01$, ***$p < 0.001$. Source data are provided as a Source Data file.

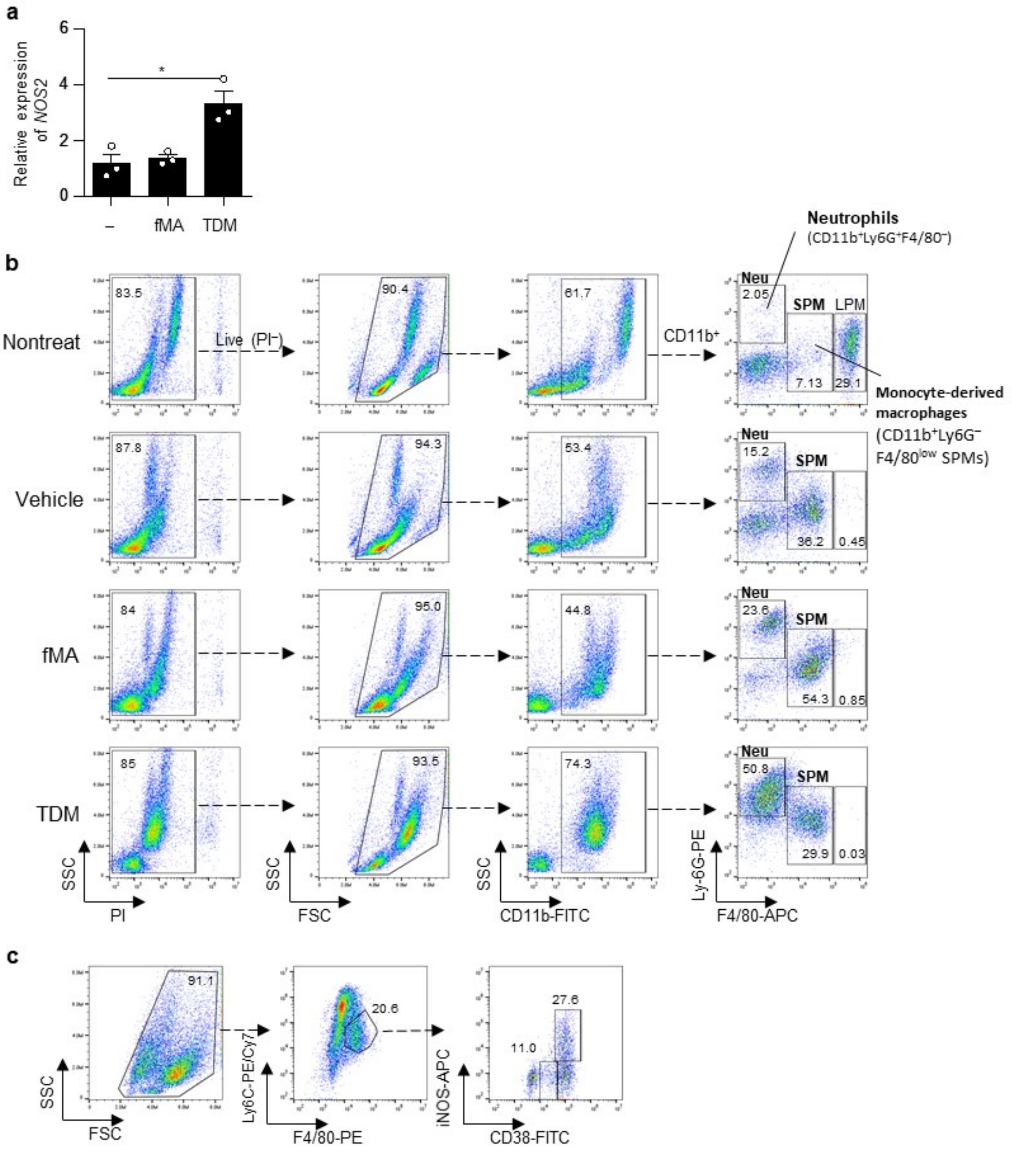

Supplementary Figure 5

iNOS expression by BMDMs and flowcytometric identification of neutrophils and macrophages recruited to the peritoneal induced by fMA and TDM. (**a**) BMDMs from C57BL/6 mice were stimulated with 1 μg/well of fMA or TDM coated on the plates in the presence of 10 ng/ml of IFNγ for 24 h. *Nos2* mRNA levels were measured by qRT-PCR. Data are presented as the mean ± SEM of triplicate assay and are representative of two independent experiments. The statistical significance was calculated by one-way ANOVA followed by Bonferroni's test. $*p < 0.05$, $**p < 0.01$, $***p < 0.001$. (**b**) Control (vehicle), MA, or

TDM emulsions were intraperitoneally injected, and the peritoneal cells were collected and analyzed by flow cytometry. Recruitment of neutrophils ($CD11b^+Ly6G^+F4/80^-$) and monocyte-derived macrophages (SPMs; $CD11b^+Ly6G^-F4/80^{low}$) was analyzed after gating propidium iodide $(PI)^-$ cells. Source data are provided as a Source Data file. (**c**) The gating strategy used for the analysis in Figure 5h.

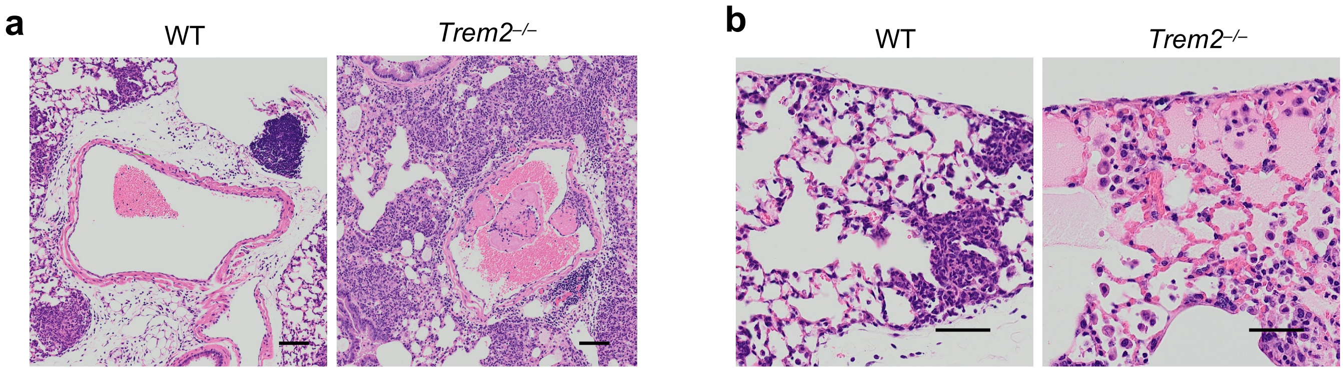

Supplementary Figure 6

Vasculitis and edema in TDM-injected *Trem2*$^{-/-}$ mice. WT or *Trem2*$^{-/-}$ mice were intravenously injected with 50 μg TDM emulsion, and the lungs were collected at day 7 after the injection. Representative hematoxylin & eosin-stained images of vasculitis (scale bars; 100 μm) (**a**) and edema (scale bars; 50 μm) (**b**) are shown. The images are representative of two independent experiments.

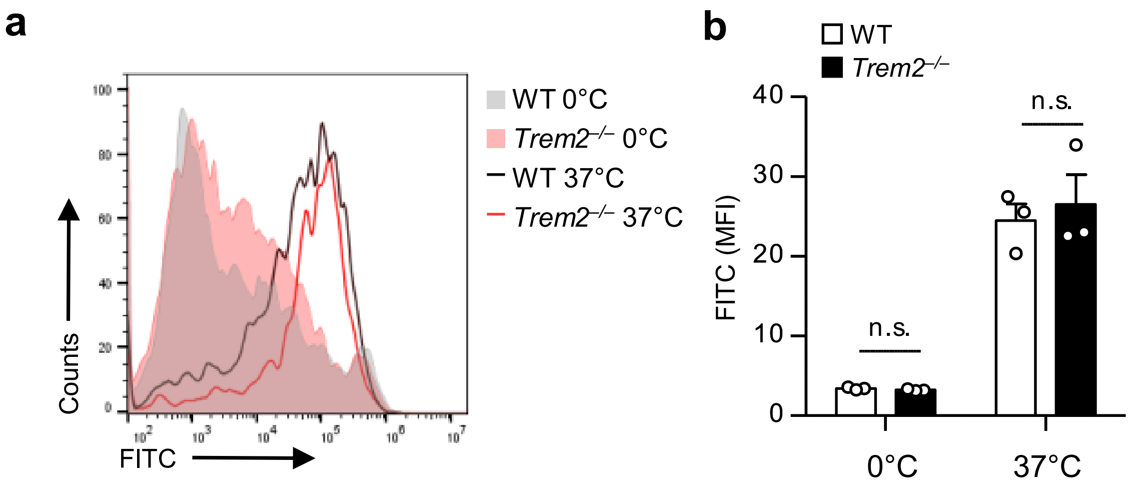

Supplementary Figure 7

TREM2 deficiency does not affect the phagocytosis of mycobacteria by BMDMs. (**a**, **b**) WT or *Trem2*$^{-/-}$ BMDMs were incubated with FITC-labeled BCG (MOI 10) at 0 or 37°C for 4 h. After quenching by trypan blue, the phagocytosed bacteria were assessed by flow cytometry (**a**). The phagocytic activity was quantified by MFI (**b**). Data are presented as the mean ± SEM of triplicate assay and are representative of three independent experiments. The statistical significance was calculated by two-tailed unpaired *t*-test. n.s. : not significant. Source data are provided as a Source Data file.

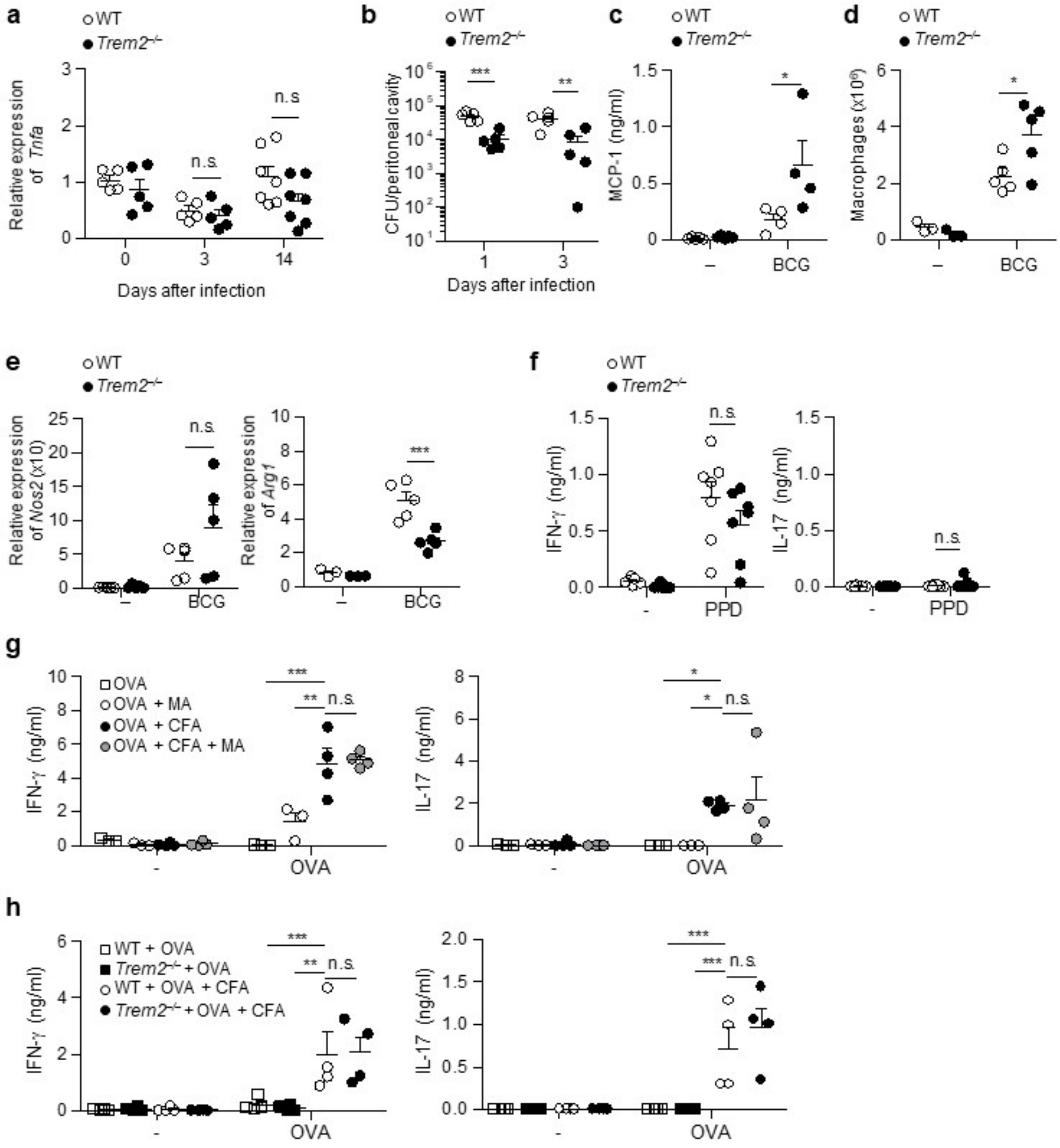

Supplementary Figure 8

TREM2 deficiency accelerates the clearance of mycobacteria. (a) The expressions of *Tnfa* in the lungs from uninfected control (n = 5) and infected mice were analyzed by qRT-PCR. Data are presented as the mean ± SEM and are representative of two independent experiments. The statistical significance was calculated by two-tailed unpaired t-test. n.s. : not significant. **b-e**) WT or *Trem2* $^{-/-}$ mice (n = 5) were infected intraperitoneally with 5.0×10^6 CFU of *M. bovis* BCG. (**b**) Peritoneal cells were collected at days 1 and 3 after infection and CFUs were determined. (**c**) At 4 h post-infection, peritoneal lavages were collected from uninfected control (–; n = 4) and infected mice (BCG; n = 5), and the level of MCP-1 was determined by ELISA. (**d, e**) Peritoneal cells (–; n = 3; BCG, n = 5) were collected at day 3 after infection. The numbers of recruited macrophages ($CD11b^+Ly6C^+F4/80^+$) were determined by flow cytometry (**d**), and *Nos2* and *Arg 1* mRNA levels were determined by qRT-PCR (**e**). Data are presented as the mean ± SEM and are representative of two independent experiments. The statistical significance was calculated by two-way ANOVA followed by

Bonferroni's test. *$p < 0.05$, **$p < 0.01$, ***$p < 0.001$.

(**f**) Mediastinal lymph node cells were collected from WT or *Trem2* $^{-/-}$ mice (n = 7) at day14 after intratracheal infection with 7.5×10^6 CFU of *M. bovis* BCG. The cells were stimulated with 5 μg/ml of PPD for 3 days. The levels of IFN-γ and IL-17A in the culture were measured by ELISA. Data are presented as the mean ± SEM and are representative of two independent experiments. The statistical significance was calculated by two-way ANOVA followed by Bonferroni's test. n.s. : not significant. (**g**) C57BL/6 mice were immunized with 100 μg of OVA in IFA (OVA, n = 3), IFA plus MA (OVA+MA, n = 3), CFA (OVA+CFA, n = 4), or CFA plus MA (OVA+CFA+MA, n = 4). At 28 days after immunization, spleen cells were collected and stimulated *ex vivo* with 50 μg/ml of OVA for 3 days. The levels of IFN-γ and IL-17A in the culture were measured by ELISA. (**h**) WT or *Trem2*$^{-/-}$ mice (n = 4) were immunized with OVA with or without CFA. At 28 days after immunization, spleen cells were collected and assessed for the production of IFN-γ and IL-17A as in (**g**). Data are presented as the mean ± SEM and are representative of two independent experiments. The statistical significance was calculated by two-way ANOVA followed by Bonferroni's test. *$p < 0.05$, **$p < 0.01$, ***$p < 0.001$, n.s. : not significant. Source data are provided as a Source Data file.

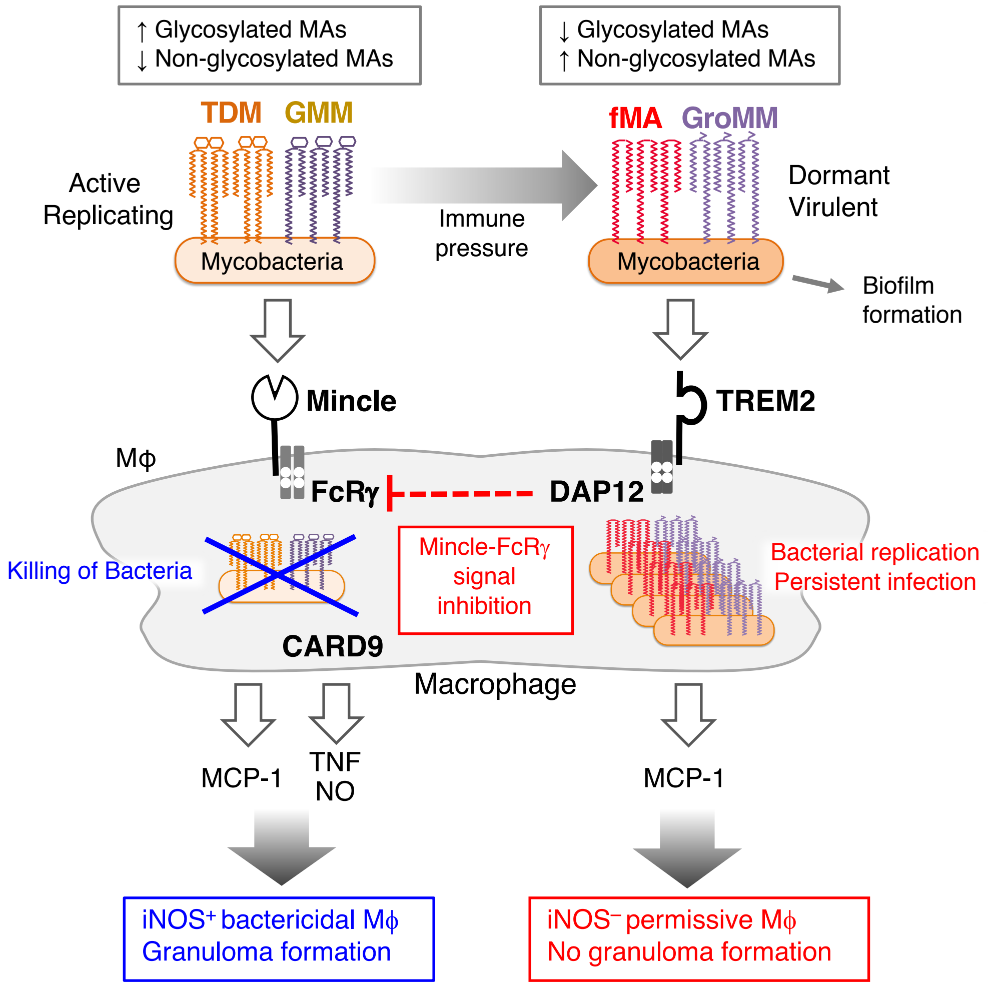

Supplementary Figure 9

A hypothesis of immune evasion of mycobacteria via TREM2. In the cell walls of replicating mycobacteria, glycosylated MA-containing lipids, such as TDM and GMM, are predominant than non-glycosylated MA-containing lipids such as free MA and GroMM. The glycosylated MAs are recognized by macrophages through Mincle and activate intracellular signals via FcRγ to produce MCP-1 as well as TNF/NO in a CARD9-dependent manner, resulting in the induction of mycobactericidal macrophages and granuloma formation that limit intracellular bacterial replication. On the other hands, in the cell walls of dormant mycobacteria or some virulent mycobacterial strains, the non-glycosylated MA-containing lipids become predominant than the glycosylated ones. The non-glycosylated MAs promote biofilm formation as well as are recognized by macrophages through TREM2, and activate intracellular signals via DAP12 to produce MCP-1 in a CARD9-independent manner without producing TNF and NO, resulting in the induction of mycobacterium-permissive macrophages without forming granulomas. In addition, the TREM2-DAP12 signaling specifically suppresses the Mincle-FcRγ signal and responses. Therefore, the TREM2-DAP12 signal activation elicited by the non-glycosylated MAs inhibits anti-mycobacterial innate immunity and may contribute to persistent infection and latency of mycobacteria in macrophages.

Supplementary Table 1. Sequences of the primers used for cloning of ITAM-coupled receptors.

Receptor	Ectodomain (a.a. number)		Sequence	Cloning site
CLEC2	23-229	Forward	5'-CTGGGGCCCAGCCGGCCGCGGAGAAGGAAAATCTCTCA-3'	*Sfi* I-*Sal* I
		Reverse	5'-GTCGACAGAAGCAGTTGGTCCACTCTTGT-3'	
Mincle	54-214	Forward	5'-CTGGGGCCCAGCCGGCCGGGCAGAACTTACAGCCACAT-3'	*Sfi* I-*Sal* I
		Reverse	5'-GTCGACAGGTCCAGAGGACTTATTTCTGG-3'	
CLEC9A	71-264	Forward	5'-CTGGGGCCCAGCCGGCCAGACTCATCCAACAGGACACA-3'	*Sfi* I-*Sal* I
		Reverse	5'-GTCGACAGGATGCAGGATCCAAATGCCTT-3'	
MGL1	156-332	Forward	5'-CTGGGGCCCAGCCGGCCTCTGATTTAACCGACCATGTA-3'	*Sfi* I-*Sal* I
		Reverse	5'-GTCGACAGTGGAACTGAGGCTATAAGTTG-3'	
SIGNR1	80-325	Forward	5'-CTGGGGCCCAGCCGGCCAATACCGAGAGGCAGAAGGAA-3'	*Sfi* I-*Xho* I
		Reverse	5'-CTCGAGCCTTCAGTGCATGGGGTTGC-3'	
SIGNR3	80-237	Forward	5'-CTGGGGCCCAGCCGGCCAGCTCAGAGGTTCAGAACAAA-3'	*Sfi* I-*Sal* I
		Reverse	5'-GTCGACAGTTTGGTGGTGCATGATGAGGT-3'	
DCAR	39-176	Forward	5'-CTGGGGCCCAGCCGGCCGACAAAGTCTGGAGCTGTTGC-3'	*Sfi* I-*Sal* I
		Reverse	5'-GTCGACAGTAAGTTTATTTTCTTCATCTG-3'	
CLEC5A	33-190	Forward	5'-CTGGGGCCCAGCCGGCCAAAAGTAATGATGGCTTCGTC-3'	*Sfi* I-*Sal* I
		Reverse	5'-GTCGACAGTTTGGCATTCATTTCGCAGAT-3'	
DC-SIGN	76-404	Forward	5'-CTGGGGCCCAGCCGGCCGCGATCTACCAGAACCTGACC-3'	*Sfi* I-*Sal* I
		Reverse	5'-GTCGACAGCGCAGGAGGGGGGTTTGGGGT-3'	
TREM1	21-195	Forward	5'-CTGGGGCCCAGCCGGCCGCCATTGTTCTAGAGGAAGAA-3'	*Sfi* I-*Sal* I
		Reverse	5'-GTCGACAGAGCATCTGTCCCATTTATGATAGT-3'	
TREM2	19-169	Forward	5'-CTGGGGCCCAGCCGGCCCTCAACACCACGGTGCTGCAG-3'	*Sfi* I-*Sal* I
		Reverse	5'-GTCGACAGGGGTGGGAAGGAGGTCTCTTGATT-3'	
TREM3	20-138	Forward	5'-CTGGGGCCCAGCCGGCCGGAGATGAGGAAGAACACAAG-3'	*Sfi* I-*Sal* I
		Reverse	5'-GTCGACAGCACCATCACTGGCTTCCCTTG-3'	
LMIR2	25-185	Forward	5'-CTGGGGCCCAGCCGGCCCTGCATGGCCCCAGCACTATC-3'	*Sfi* I-*Sal* I
		Reverse	5'-GTCGACAGGCTGCTCCGAAGAGACCGAGG-3'	
LMIR4	19-174	Forward	5'-CTGGGGCCCAGCCGGCCCAGGATTCAGTCACAGGTCCA-3'	*Sfi* I-*Sal* I
		Reverse	5'-GTCGACACGCTCAGCAGGGACCTGGTGTG-3'	
LMIR5	65-177	Forward	5'-CTGGGGCCCAGCCGGCCCAAGGCCCAGCATTGGTGAGG-3'	*Sfi* I-*Sal* I
		Reverse	5'-AGTCGACATGGTATCTTTACCCAC-3'	
LMIR7	19-180	Forward	5'-CTGGGGCCCAGCCGGCCCAGGATCCAGTCACAGGTCCA-3'	*Sfi* I-*Sal* I
		Reverse	5'-GTCGACAGGAAGAGAGAGTTCTGAGTCAC-3'	
LMIR8	22-188	Forward	5'-CTGGGGCCCAGCCGGCCCATTTCCCTGTGCGTGGTCCC-3'	*Sfi* I-*Sal* I
		Reverse	5'-GTCGACAGGAGGGGGCTGCTCAGAAGAGA-3'	

Supplementary Table 2. Sequences of the primers used for qRT-PCR.

Transcript		Sequence
Gapdh	Forward	5'-AACTTTGGCATTGTGGAAGG-3'
	Reverse	5'-ACACATTGGGGGTAGGAACA-3'
Nos2	Forward	5'-TTGGGTCTTGTTCACTCCACGG-3'
	Reverse	5'-CCTCTTTCAGGTCACTTTGGTAGG-3'
Ccl2	Forward	5'-GTTGGCTTCAGCCAGATGCA-3'
	Reverse	5'-AGCCTACTCATTGGGATCATCTTG-3'
Adgre1	Forward	5'-GTGACTCACCTTGTGGTCCT-3'
	Reverse	5'-CAGACACTCATCAACATCTGCG-3'
Arg1	Forward	5'-ACCTGGCCTTTGTTGATGTCCCTA-3'
	Reverse	5'-AGAGATGCTTCCAACTGCCAGACT-3'
