# Supplementary material for: TREM2 is a receptor for non-glycosylated mycolic acids of mycobacteria that limits anti-mycobacterial macrophage activation
Source: Nat Commun. 2021 Apr 16;12:2299. doi: 10.1038/s41467-021-22620-3 (PMC8052348; doi:10.1038/s41467-021-22620-3)
Supplement: Supplementary file 2 — Reporting Summary [file 41467_2021_22620_MOESM2_ESM.pdf]

nature research

Corresponding author(s): Hiromitsu Hara

Last updated by author(s): Mar 15, 2021

Reporting Summary

Nature Research wishes to improve the reproducibility of the work that we publish. This form provides structure for consistency and transparency in reporting. For further information on Nature Research policies, see our Editorial Policies and the Editorial Policy Checklist.

Statistics

For all statistical analyses, confirm that the following items are present in the figure legend, table legend, main text, or Methods section.

n/a	Confirmed	
☐	☒	The exact sample size (n) for each experimental group/condition, given as a discrete number and unit of measurement
☐	☒	A statement on whether measurements were taken from distinct samples or whether the same sample was measured repeatedly
☐	☒	The statistical test(s) used AND whether they are one- or two-sided<br>*Only common tests should be described solely by name; describe more complex techniques in the Methods section.*
☒	☐	A description of all covariates tested
☐	☒	A description of any assumptions or corrections, such as tests of normality and adjustment for multiple comparisons
☐	☒	A full description of the statistical parameters including central tendency (e.g. means) or other basic estimates (e.g. regression coefficient) AND variation (e.g. standard deviation) or associated estimates of uncertainty (e.g. confidence intervals)
☐	☒	For null hypothesis testing, the test statistic (e.g. F, t, r) with confidence intervals, effect sizes, degrees of freedom and P value noted<br>*Give P values as exact values whenever suitable.*
☒	☐	For Bayesian analysis, information on the choice of priors and Markov chain Monte Carlo settings
☒	☐	For hierarchical and complex designs, identification of the appropriate level for tests and full reporting of outcomes
☒	☐	Estimates of effect sizes (e.g. Cohen's d, Pearson's r), indicating how they were calculated

Our web collection on statistics for biologists contains articles on many of the points above.

Software and code

Policy information about availability of computer code

Data collection	Flow cytometory: CytExpert V2.3 (using CytofLEX)<br>Microplate reader: SoftMaxPro (using VERSAmax micro plate reader)<br>Microscope: BZ-H4XD (using All-in-One BZ-X700)<br>qPCR: StepOne Software V2.3 (using StepOne Plus)
Data analysis	FACS analysis: FlowJo V10.5.3<br>Statistical analysis: GraphPad Prism V5

For manuscripts utilizing custom algorithms or software that are central to the research but not yet described in published literature, software must be made available to editors and reviewers. We strongly encourage code deposition in a community repository (e.g. GitHub). See the Nature Research guidelines for submitting code & software for further information.

Data

Policy information about availability of data

All manuscripts must include a data availability statement. This statement should provide the following information, where applicable:

- Accession codes, unique identifiers, or web links for publicly available datasets
- A list of figures that have associated raw data
- A description of any restrictions on data availability

The data that support the finding in this study are available from the corresponding author upon reasonable request. Source data are provided with this paper.

Field-specific reporting

Please select the one below that is the best fit for your research. If you are not sure, read the appropriate sections before making your selection.

[x] Life sciences [ ] Behavioural & social sciences [ ] Ecological, evolutionary & environmental sciences

For a reference copy of the document with all sections, see nature.com/documents/nr-reporting-summary-flat.pdf

Life sciences study design

All studies must disclose on these points even when the disclosure is negative.

Sample size	Sample sizes are indicated in the manuscript. Statistical methods were not used to determine the sample size. We determined it based on our prior study (Hara H. et al., Nat. immunol., 8, 619-629, 2007; Yasukawa S. et al., Nat. commun., 5, 3755, 2014.).
Data exclusions	No data were excluded.
Replication	As reported in the manuscript, all experiments were repeated at least twice.
Randomization	Mice were randomly assigned to each experimental group in all animal experiments.
Blinding	In animal and cell culture experiments, blinding was not necessary because measurements were empirical and not subjective. In the qualitative analysis, such as pathological analysis, which prejudice may lead to bias, the data were evaluated by the investigator blinded to experimental group allocations.

Reporting for specific materials, systems and methods

We require information from authors about some types of materials, experimental systems and methods used in many studies. Here, indicate whether each material, system or method listed is relevant to your study. If you are not sure if a list item applies to your research, read the appropriate section before selecting a response.

Materials & experimental systems

n/a	Involved in the study	
[ ]	[x]	Antibodies
[ ]	[x]	Eukaryotic cell lines
[x]	[ ]	Palaeontology and archaeology
[ ]	[x]	Animals and other organisms
[x]	[ ]	Human research participants
[x]	[ ]	Clinical data
[x]	[ ]	Dual use research of concern

Methods

n/a	Involved in the study	
[x]	[ ]	ChIP-seq
[ ]	[x]	Flow cytometry
[x]	[ ]	MRI-based neuroimaging

Antibodies

Antibodies used	An antibody used for plate-coated lipid binding assay:<br>HRP-conjugated anti-human IgG antibody (Abcam ab6759) dilution 1:100000<br><br>Antibodies used for neutralization of TNF in the experiment of NO production:<br>Anti-mouse TNF-α antibody (MP6-XT22, Biolegend 506331) dilution 1:1000<br>Rat IgG1 κ Isotype control antibody (RTK2071, Biolegend 400457) dilution 1:1000<br><br>Antibodies used for flow cytometry:<br>FITC-conjugated anti-human/mouse CD11b antibody (M1/70, eBioscience 11-0112-41) dilution 1:20<br>PE-conjugated anti-mouse Ly6G antibody (1A8, eBioscience 12-9668-82) dilution 1:100

APC-conjugated anti-mouse F4/80 antibody (BM8.1, TONBO biosciences 20-4801) dilution 1:100
PE-conjugated anti-mouse F4/80 antibody (BM8, Biolegend 123109) dilution 1:100
FITC-conjugated anti-mouse CD38 antibody (90, Biolegend 102705) dilution 1:100
PE/Cy7-conjugaetd anti-mouse Ly6C antibody (HK1.4, Biolegend 128017) dilution 1: 333
APC-conjugated anti-mouse Nos2 antibody (CXNFT, eBioscience 17-5920-80) dilution 1:100
Anti-mouse CD16/32 antibody (2.4G2, TONBO biosciences 70-0161) dilution 1:100
FITC-conjugated anti-human IgG antibody (Jackson ImmunoResearch 709-095-149) dilution 1:200

Antibodies used for MACS:
PE-conjugated anti-rat CD2 antibody (OX-34, Biolegend 210315) dilution 1:100
APC-conjugated anti-human CD8 antibody (HIT8a, Biolegend 300911) dilution 1:100

Validation

All antibodies used in this study have been previously validated by the manufacturers.

An antibody used for plate-coated lipid binding assay:
HRP-conjugated anti-human IgG antibody (Abcam ab6759, https://www.abcam.co.jp/rabbit-human-igg-hl-hrp-ab6759.html)

Antibodies used for neutralization of TNF in the experiment of NO production:
Anti-mouse TNF-α antibody (MP6-XT22, Biolegend 506331, https://www.biolegend.com/ja-jp/products/ultra-leaf-purified-anti-mouse-tnf-alpha-antibody-7753); Rat IgG1 κ Isotype control antibody (RTK2071, Biolegend 400457, https://www.biolegend.com/ja-jp/products/ultra-leaf-purified-rat-igg1-kappa-isotype-ctrl-7725)

Antibodies used for flow cytometry:
FITC-conjugated anti-human/mouse CD11b antibody (M1/70, eBioscience 11-0112-41, https://www.thermofisher.com/antibody/product/CD11b-Antibody-clone-M1-70-Monoclonal/11-0112-41); PE-conjugated anti-mouse Ly6G antibody (1A8, eBioscience 12-9668-82, https://www.thermofisher.com/antibody/product/Ly-6G-Antibody-clone-1A8-Ly6g-Monoclonal/12-9668-82); APC-conjugated anti-mouse F4/80 antibody (BM8.1, TONBO biosciences 20-4801, https://tonbobio.com/products/apc-anti-mouse-f4-80-antigen-bm8-1); PE-conjugated anti-mouse F4/80 antibody (BM8, Biolegend 123109, https://www.biolegend.com/ja-jp/products/pe-anti-mouse-f4-80-antibody-4068); FITC-conjugated anti-mouse CD38 antibody (90, Biolegend 102705, https://www.biolegend.com/ja-jp/products/fitc-anti-mouse-cd38-antibody-182); PE/Cy7-conjugaetd anti-mouse Ly6C antibody (HK1.4, Biolegend 128017, https://www.biolegend.com/ja-jp/products/pe-cyanine7-anti-mouse-ly-6c-antibody-6063); APC-conjugated anti-mouse Nos2 antibody (CXNFT, eBioscience 17-5920-80, https://www.thermofisher.com/antibody/product/iNOS-Antibody-clone-CXNFT-Monoclonal/17-5920-82); Anti-mouse CD16/32 antibody (2.4G2, TONBO biosciences 70-0161, https://tonbobio.com/products/purified-anti-mouse-cd16-cd32-2-4g2-fc-block); FITC-conjugated anti-human IgG antibody (Jackson ImmunoResearch 709-095-149, https://www.jacksonimmuno.com/catalog/products/709-095-149)

Antibodies used for MACS:
PE-conjugated anti-rat CD2 antibody (OX-34, Biolegend 210315, https://www.biolegend.com/ja-jp/products/pe-anti-rat-cd2-antibody-2379); APC-conjugated anti-human CD8 antibody (HIT8a, Biolegend 300911, https://www.biolegend.com/ja-jp/products/apc-anti-human-cd8a-antibody-759)

Eukaryotic cell lines

Policy information about cell lines

Cell line source(s)

Freestyle 293 cells were purchased from Thermo fisher scientific. 2B4 NFAT-GFP and Phoenix cells were kindly provided by Dr. Takashi Saito (RIKEN).

Authentication

Non of the cell lines used in this study were authenticated.

Mycoplasma contamination

No mycoplasma contamination.

Commonly misidentified lines
(See ICLAC register)

No misidentified cell lines were used in this study.

Animals and other organisms

Policy information about studies involving animals; ARRIVE guidelines recommended for reporting animal research

Laboratory animals

All male and female mice in this study were between ages of 8-15 weeks. Mice were maintained in a specific pathogen free unit on a 12hr light/12hr dark cycle. Room temperature was maintained at 25°C. The humidity level was controlled between 40-60%.

The following strains were used:
C57BL/6N WT mice
Trem2 -/- mice
Clec4e -/- mice
Card9 -/- mice
Tyrobp -/- mice
Fcer1g -/- mice

Wild animals	No wild animals were used in this study.
Field-collected samples	No field-collected samples were used in this study.
Ethics oversight	All animal experiments have been approved by the Institutional Animal Research Committee of Kagoshima University and animals were treated in accordance with the ethical guidelines of Kagoshima University.

Note that full information on the approval of the study protocol must also be provided in the manuscript.

Flow Cytometry

Plots

Confirm that:

- [x] The axis labels state the marker and fluorochrome used (e.g. CD4-FITC).
- [x] The axis scales are clearly visible. Include numbers along axes only for bottom left plot of group (a 'group' is an analysis of identical markers).
- [x] All plots are contour plots with outliers or pseudocolor plots.
- [x] A numerical value for number of cells or percentage (with statistics) is provided.

Methodology

Sample preparation	2B4 NFAT-GFP reporter cells were stimulated in 96 wells and collected by pipetting. The infiltrated cells in the peritoneal lavage were collected by washing the peritoneum cavity with RPMI-10. The cell were then stained by the antibodies as described in the manuscript.
Instrument	CytoFLEX
Software	FlowJo V10.5.3
Cell population abundance	While acquiring data for the analysis, at least 5,000 to 10,000 cells were included in stopping gate.
Gating strategy	Gating strategy for each experiment is provided in the manuscript.

- [x] Tick this box to confirm that a figure exemplifying the gating strategy is provided in the Supplementary Information.
